# Synthesis and crystal structures of five fluorinated diphenidine derivatives

**DOI:** 10.1107/S2056989025001288

**Published:** 2025-02-14

**Authors:** Ryan E. Mewis, Matthew C. Hulme, Jack Marron, Stuart K. Langley, Oliver B. Sutcliffe, Sophie L. Benjamin

**Affiliations:** a Department of Natural Sciences, Manchester Metropolitan University, John Dalton Building, Chester St., Manchester, M1 5GD, United Kingdom; b School of Science and Technology, Nottingham Trent University, Nottingham NG11, 8NS, United Kingdom; Universidade Federal do ABC, Brazil

**Keywords:** crystal structure, diphenidine, novel psychoactive substances

## Abstract

Diphenidine (**1a**), a dissociative anaesthetic, was first reported in 2013. Since then, a number of derivatives *e.g.* 2-methoxphenidine (**1b**) have been produced by clandestine laboratories and sold as *research chemicals*. Fluorinated diphenidines, namely, [1-(2,6-di­fluoro­phen­yl)-2-phenyl­eth­yl]di­methyl­aza­nium chloride, C_16_H_18_F_2_N^+^·Cl^−^, (**I**), [1-(2,6-di­fluoro­phen­yl)-2-phenyl­eth­yl](eth­yl)aza­nium chloride di­chloro­methane hemisolvate, 2C_16_H_18_F_2_N^+^·2Cl^−^·CH_2_Cl_2_, (**II**), *tert*-but­yl[1-(2,6-di­fluoro­phen­yl)-2-phenyl­eth­yl]aza­nium chloride, C_18_H_22_F_2_N^+^·Cl^−^, (**III**), 1-[1-(2,6-di­fluoro­phen­yl)-2-phenyl­eth­yl]pyrrolidin-1-ium chloride, C_18_H_20_F_2_N^+^·Cl^−^, (**IV**), and 1-[1-(2,3,4,5,6-penta­fluoro­phen­yl)-2-phenyl­eth­yl]piperidin-1-ium chloride, C_19_H_19_F_5_N^+^·Cl^−^, (**V**), were synthesized and structurally characterized by ^1^H, ^13^C and ^19^F NMR spectroscopy, and single-crystal X-ray diffraction. All five structures exhibit hydrogen bonding between the quaternary amine hydrogen atoms and the chlorine. The N—H⋯Cl distances for (**II**) and (**III**) range from 2.21 to 2.31 Å, whereas (**I**), (**IV**) and (**V**) exhibit shorter N—H⋯Cl distances (2.07–2.20 Å). Compounds (**IV**) and (**V**) include pyrrolidine and piperidine rings, respectively; the pyrrolidine ring adopts an envelope conformation whereas the piperidine ring adopts a chair conformation. The crystal packing in compounds (**I**)–(**V**) is characterized by C—H⋯π inter­actions; no π–π inter­actions are observed.

## Abstract 2

The crystal structures of five fluorinated diphenidine mol­ecules obtained as their hydro­chloride salts are reported.

## 1. Chemical context

Over the past two decades, there has been a significant increase in the number of new psychoactive substances (NPS) seized by law enforcement agencies globally (King, 2013 ▸; UNODC, 2024 ▸). Current convention uses a functional ‘effect group’ categorization to define NPS within six broad overlapping groups: (i) synthetic cannabinoid receptor agonists; (ii) classic hallucinogens; (iii) stimulants; (iv) opioid receptor agonists; (v) sedatives/hypnotics and (vi) dissociatives (UNODC, 2024 ▸; Tettey *et al.*, 2018 ▸; Shafi *et al.*, 2020 ▸). NPS are assigned to a specific ‘effect group’ based on their chemical structure and psychopharmacological effects (UNODC, 2024 ▸; Tettey *et al.*, 2018 ▸). 1,2-Di­aryl­ethamines are dissociative, psychoactive substances, which distort perceptions, produce feelings of detachment, and induce a state of anaesthesia by antagonizing ionotropic *N*-methyl-d-aspartate receptors (NMDAR) in the central nervous system (UNODC, 2024 ▸; Morris & Wallach, 2014 ▸).

The first of these dissociative anaesthetics was 1-(1,2-di­phenyl­eth­yl)piperidine (diphenidine, **1a**) (Wallach *et al.*, 2015 ▸) reported in 2013 (Morris & Wallach, 2014 ▸), followed by 1-[1-(2-meth­oxy­phen­yl)-2-phenyl­eth­yl]piperidine (2-methox­phen­idine, **1b**) (McLaughlin *et al.*, 2016 ▸), which have both been marketed as ‘*research chemicals*’ and encountered in tablet or powder forms (UNODC, 2024 ▸; Wallach *et al.*, 2015 ▸; McLaughlin *et al.*, 2016 ▸; Odoardi *et al.*, 2016 ▸; Strano Rossi *et al.*, 2014 ▸) or in combination with synthetic cannabinoids such as AB-CHMINACA, 5F-AMB (Hasegawa *et al.*, 2015 ▸) and 5F-AB-PINACA (Wurita *et al.*, 2014 ▸). Though both the supply and production of **1a**, **1b** and the recently disclosed 1-[1-(2-chloro­phen­yl)-2-phenyl­eth­yl]piperidine (2-chloro­diphenidine, **1c**) (Wallach *et al.*, 2016 ▸; Sahai *et al.*, 2018 ▸), are now controlled in the United Kingdom by the 2016 Psychoactive Substances Act (Reuter & Pardo, 2017 ▸), the emergence of novel 1,2-di­aryl­ethyl­amine derivatives, such as the fluorinated compounds, (**I**)–(**V**), still raises considerable legal and analytical challenges in both the forensic identification and discrim­ination of these materials. This is due to the inference of diphenidine-based NPS in several fatalities in Europe (Morris & Wallach, 2014 ▸; Wallach *et al.*, 2015 ▸, 2016 ▸; McLaughlin *et al.*, 2016 ▸; Strano Rossi *et al.*, 2014 ▸; Hasegawa *et al.*, 2015 ▸; Wurita *et al.*, 2014 ▸; Sahai *et al.*, 2018 ▸; Reuter & Pardo, 2017 ▸; Elliott *et al.*, 2015 ▸; Helander *et al.*, 2015 ▸; Hofer *et al.*, 2014 ▸), Asia (Hasegawa *et al.*, 2015 ▸; Minakata *et al.*, 2016 ▸; Kudo *et al.*, 2015 ▸) and **1a** being placed under inter­national control, within schedule II of the United Nations Convention on Psychotropic Substances (1971), on 14th April 2021 (UNODC, 2021 ▸).
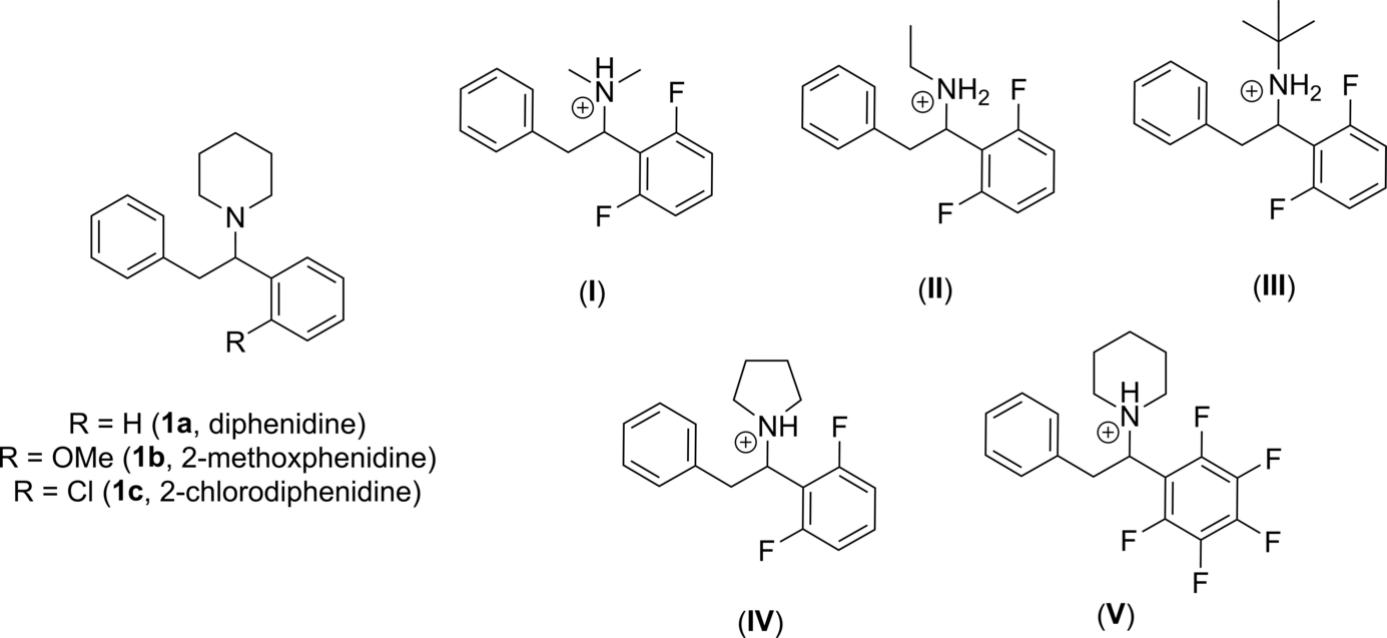


## 2. Structural commentary

**Figure 1:**
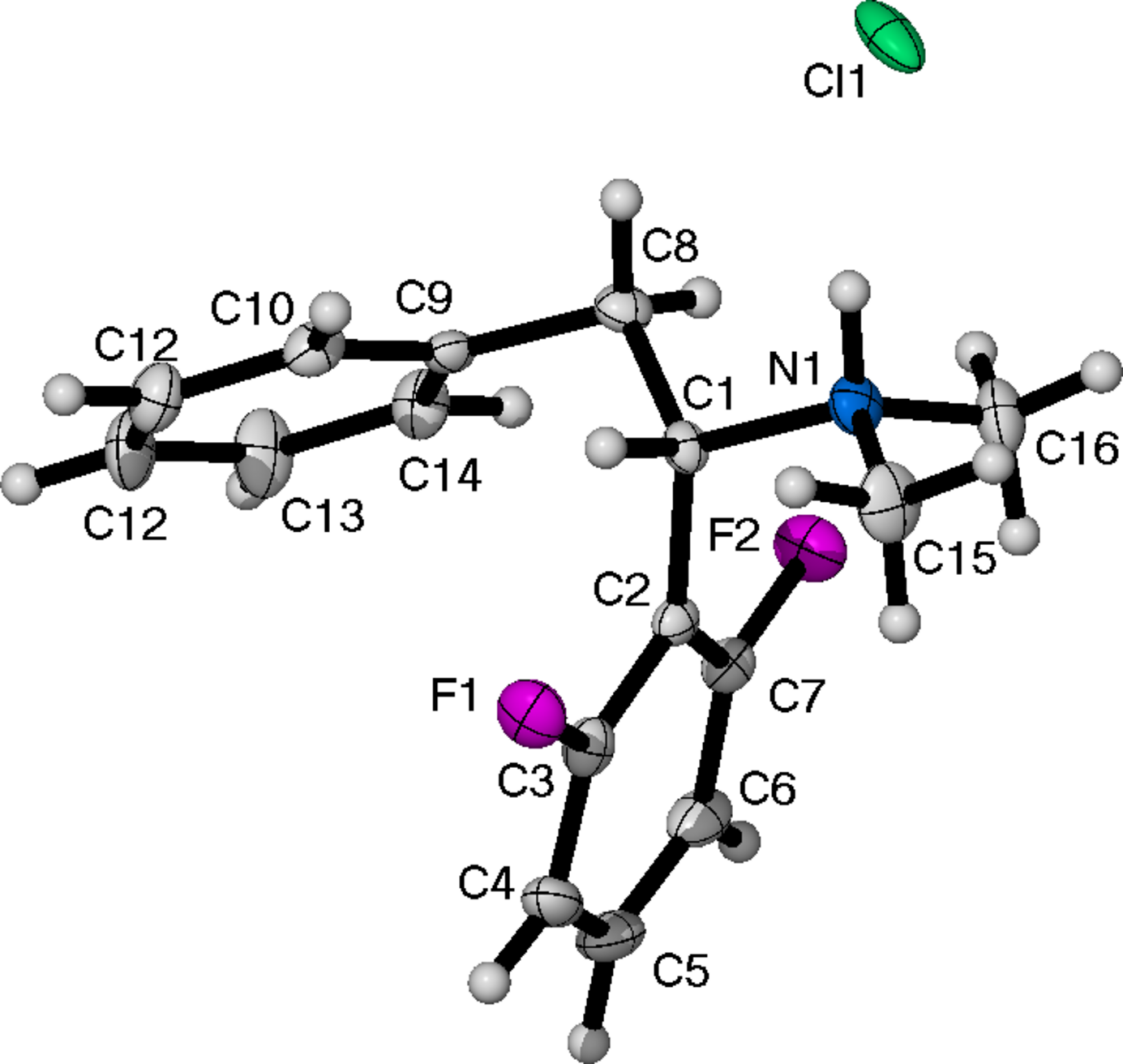
The mol­ecular structure of (**I**), showing the atom-labelling scheme and displacement ellipsoids at the 50% probability level.

Compound (**I**) (Fig. 1 ▸) crystallizes in the monoclinic space group *P*2_1_/*c* with a single mol­ecule in the asymmetric unit. The torsion angle between the two quaternary carbons of the phenyl rings and the bridging ethyl chain is 53.4 (2)°.

**Figure 2:**
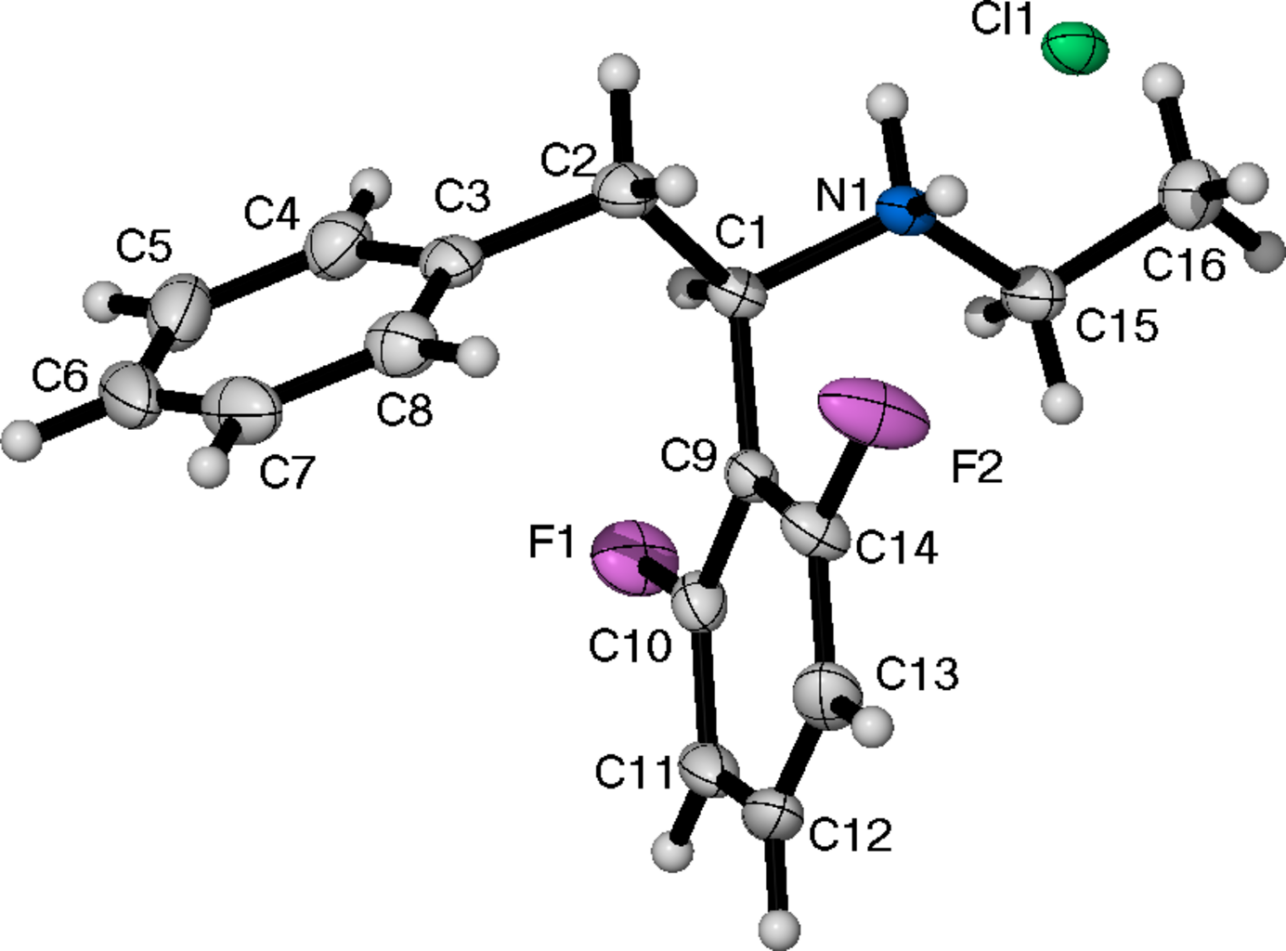
The mol­ecular structure of (**II**), showing the atom-labelling scheme and displacement ellipsoids at the 50% probability level. The half mol­ecule of DCM present has been omitted.

**Figure 3:**
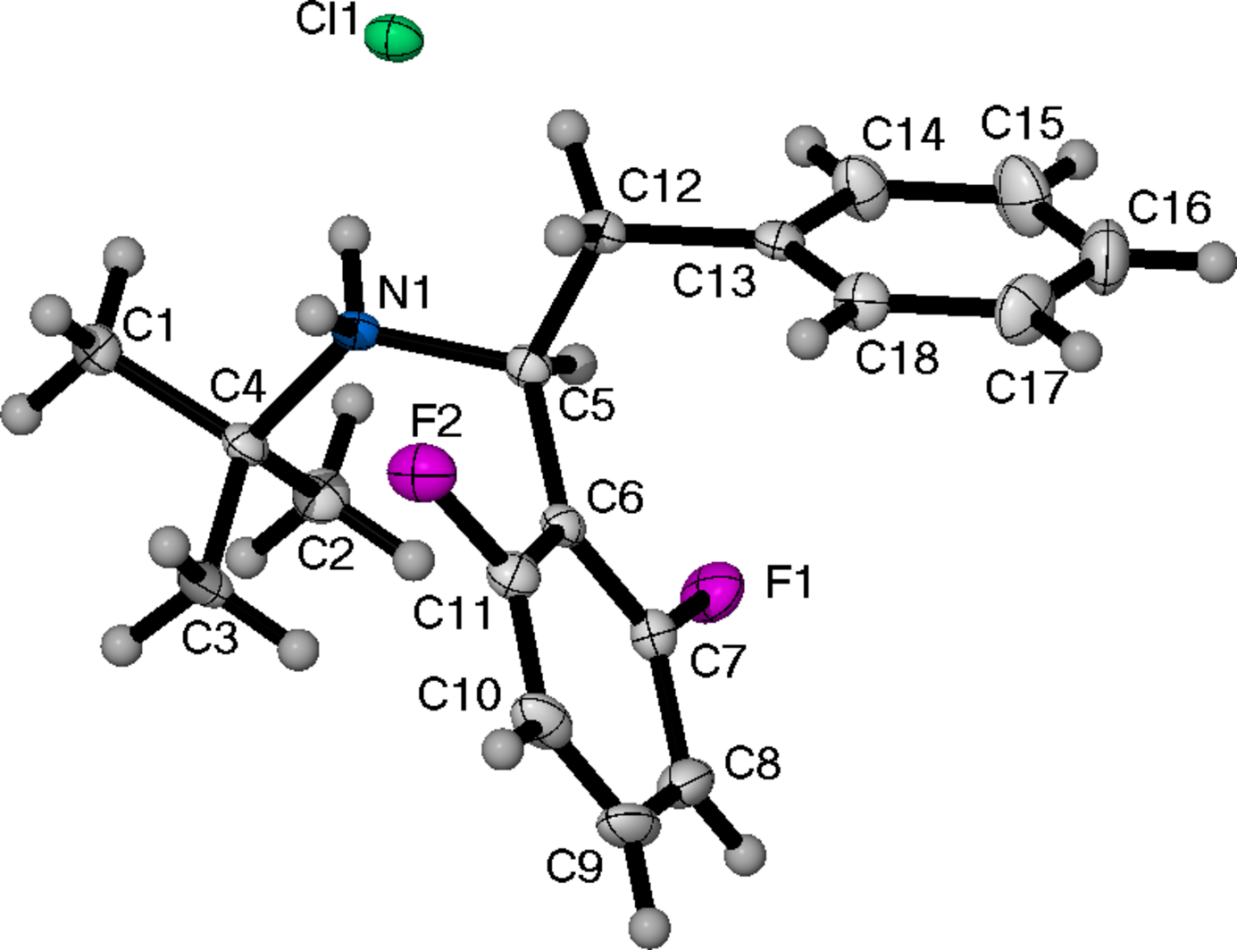
The mol­ecular structure of (**III**), showing the atom-labelling scheme and displacement ellipsoids at the 50% probability level.

Compound (**II**) (Fig. 2 ▸) crystallizes in the *I*2*/a* space group. It consists of one mol­ecule in the asymmetric unit, as well as half of a single mol­ecule of di­chloro­methane (DCM). The terminal carbon of the ethyl group (C15, C15*A*) is disordered over two positions [0.707 (5):0.293 (5) occupancy]. The closest contact between one of the fluorine atoms of the 2,6-di­fluoro­phenyl ring and a hydrogen atom of DCM is 2.335 Å. The torsion angle for (**II**), as defined previously for (**I**), is −55.9 (2)°. The final non-cyclic aliphatic analogue, (**III**) (Fig. 3 ▸), crystallizes in the monoclinic space group *P*2_1_/*c* with a single formula unit in the asymmetric unit cell. The torsion angle is the largest of all the structures presented herein at 63.8 (2)°.

**Figure 4:**
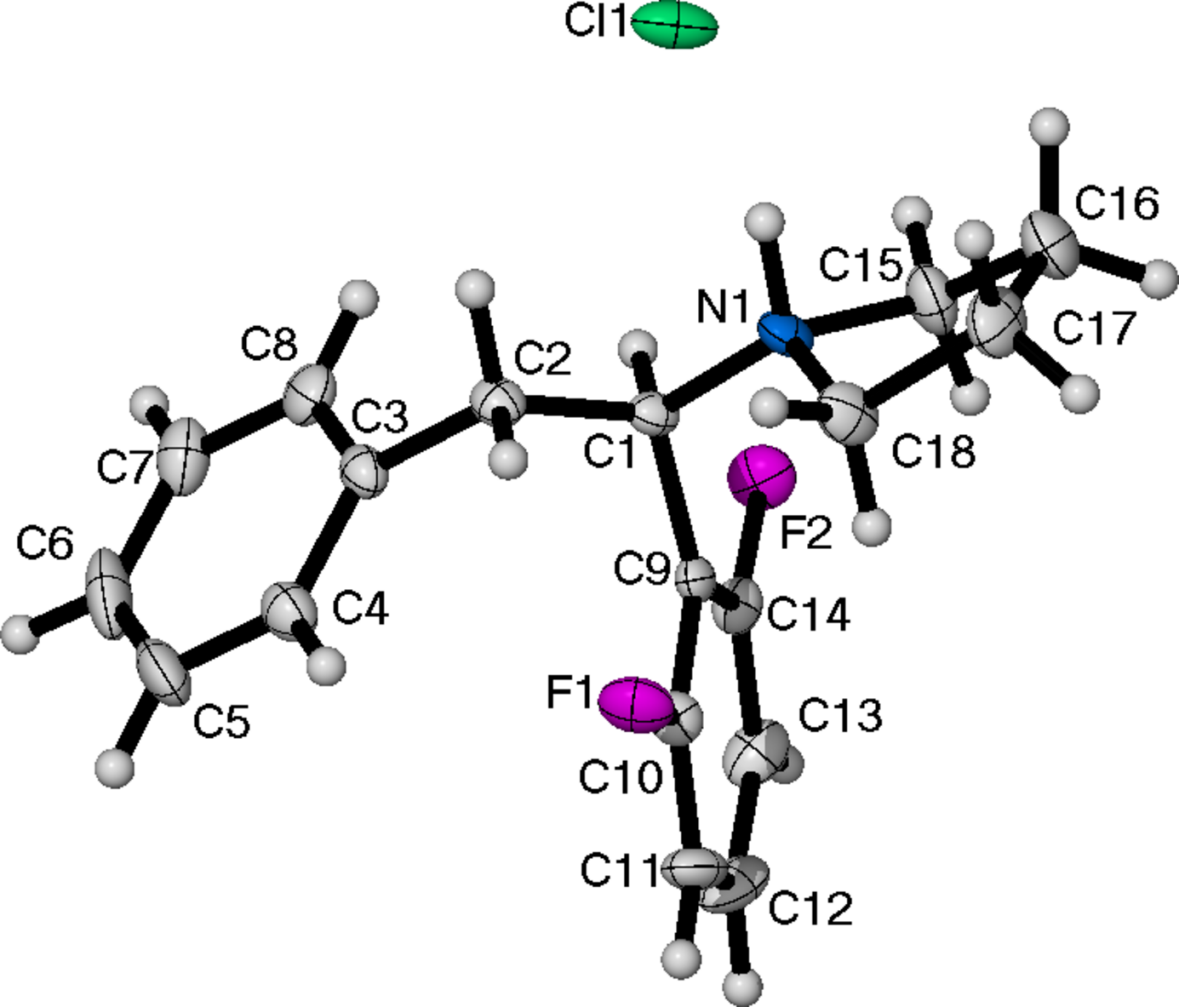
The mol­ecular structure of (**IV**), showing the atom-labelling scheme and displacement ellipsoids at the 50% probability level. Only one mol­ecule present in the asymmetric unit is shown.

Compound (**IV**) (Fig. 4 ▸) crystallizes in the triclinic space group *P*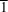 with two mol­ecules in the asymmetric unit. Torsion angles of −54.6 (3) and 58.9 (3)° are very similar to (**I**) and (**III**). The pyrrolidine ring present in the structure adopts an envelope conformation.

**Figure 5:**
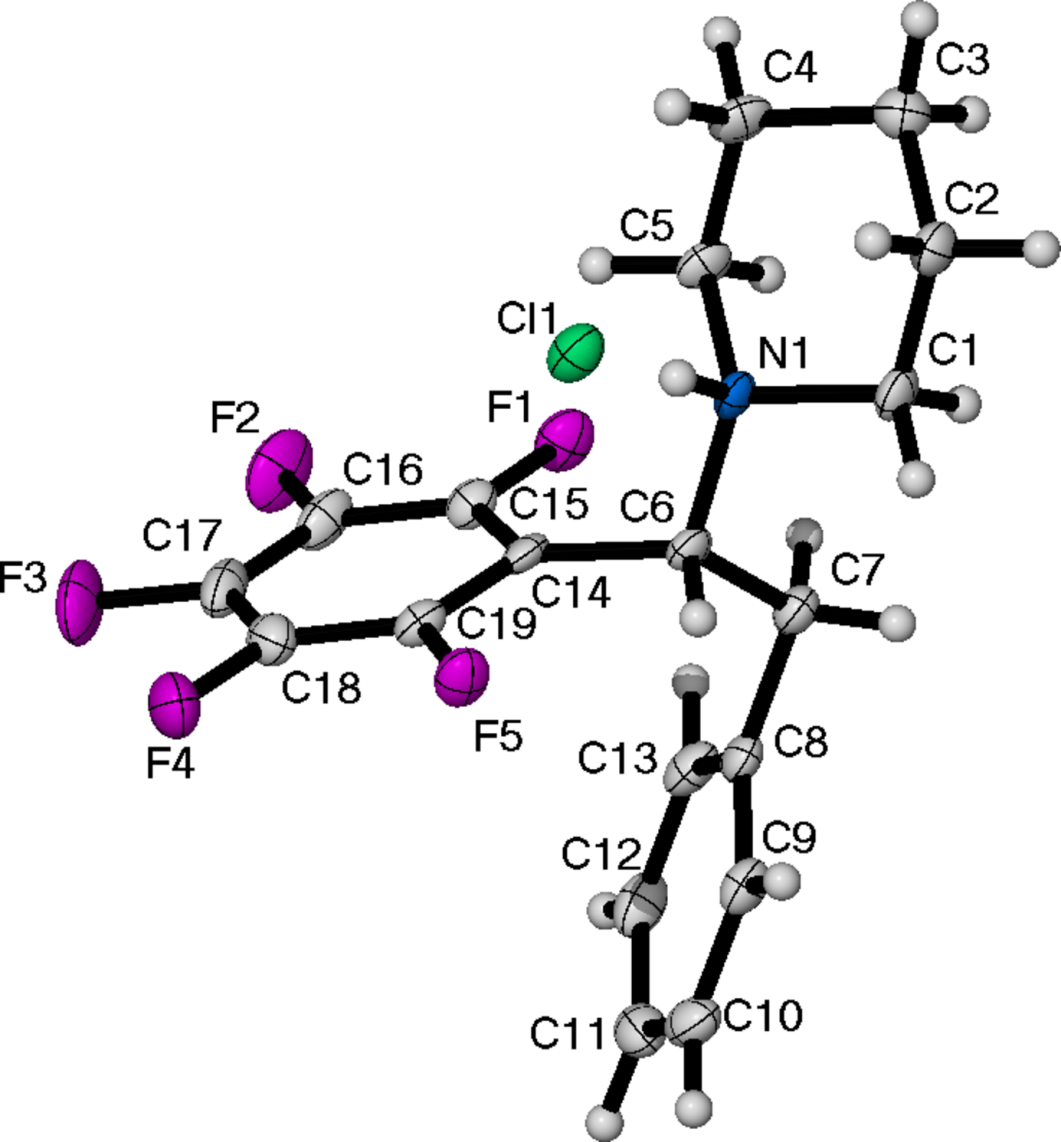
The mol­ecular structure of (**V**), showing the atom-labelling scheme and displacement ellipsoids at the 50% probability level.

Compound (**V**) (Fig. 5 ▸) crystallizes in the monoclinic space group *P*2_1_/*c* with a single mol­ecule in the asymmetric unit. The torsion angle defined is the smallest of the crystal structures presented at 47.3 (2)°. The piperidine ring is in the chair conformation. All five structures exhibit hydrogen bonding between the quaternary amine and the chlorine (Tables 1 ▸–5 ▸ ▸ ▸ ▸). The five structures can be split in to two groups; (**II**) and (**III**) both have two *R* groups attached to the amine whereas the remainder all possess three. The N—H⋯Cl distance for the former grouping range from 2.21 to 2.31 Å, with N—H—Cl angles of 151–168° (Tables 2 ▸ and 3 ▸). Inter­estingly, in (**II**), a shorter N—H1*A*⋯Cl distance of 2.11 Å (compared to 2.30 Å for N—H1*B*⋯Cl) is observed to a symmetry-related [symmetry code: (i) 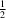 − *x*, 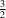 − *y*, 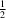 − *z*] Cl atom. The latter group, consisting of (**I**), (**IV**) and (**V**) exhibit shorter N—H⋯Cl distances (2.07–2.20 Å, Tables 1 ▸, 4 ▸ and 5 ▸) as well as N—H—Cl angles that are all greater than 163°.

## 3. Supra­molecular features

Mol­ecules of (**I**) exhibit no π–π inter­actions, as despite the unsubstituted phenyl rings being aligned when viewed along the *c*-axis direction, the shortest centroid–centroid distance is 7.947 Å [symmetry operation 1 + *x*, *y*, *z*]. Mol­ecules are linked together by C—H⋯π inter­actions; the distance of the centroid of the unsubstituted phenyl ring to the nearest aromatic protons of a substituted aromatic ring are 3.274 and 3.951 Å [*Cg*1⋯H4^i^ = 3.274 Å and *Cg*1⋯H5^i^ = 3.951 Å; *Cg*1 is the centroid of C9-C14 ring; symmetry code: (i) 1 + *x*, *y*, *z*]. Another C—H⋯π inter­action exists between the centroid of the difluorinated ring and a phenyl ring proton of a neighbouring mol­ecule [*Cg*2⋯H11^ii^ = 2.982 Å; *Cg*2 is the centroid of the C2–C7 ring; symmetry code: (ii) 1 − *x*, *y* − 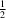, 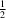 − *z*].

Analysis of (**II**)–(**V**) reveals that these also exhibit no π–π inter­actions. Similarly to (**I**), they do exhibit weak C—H⋯π inter­actions with distances of 3.244–3.425, 3.427–3.744 and 2.929–3.459 Å for (**II**), (**III**) and (**IV**), respectively. between the nearest ring hydrogen of the difluorinated ring and that of the centroid of the nearest neighbouring phenyl ring. (**II**) also exhibits a C—H⋯π inter­action between the non-fluorinated phenyl rings of neighbouring mol­ecules [*Cg*3⋯H4^iii^ = 2.969 Å; *Cg*3 is the centroid of ring C3–C8; symmetry code: (iii) −*x*, *y* − 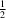, 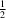 − *z*]. Similarly, (**III**) has the same inter­action [*Cg*4⋯H17^iv^ = 3.785 Å and *Cg*4⋯H18^iv^ = 4.105 Å; *Cg*4 is the centroid of ring C13–C18; symmetry code: (iv) −*x*, *y* − 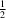, *z* − 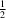]. For (**IV**), the pyrrolidine ring exhibits two sets of C—H⋯π inter­actions to the phenyl [*Cg*5⋯H17*A*^v^ = 3.349 Å and *Cg*5⋯H18*A*^v^ = 3.417 Å; *Cg*5 is the centroid of ring C27–C32; symmetry code: (v) 1 − *x*, 2 − *y*, 1 − *z*] and difluorinated rings [*Cg*6⋯H33*A*^vi^ = 4.179 Å and *Cg*6⋯H33*B*^vi^ = 4.068 Å; *Cg*6 is the centroid of ring C27–C32; symmetry code: (vi) 1 − *x*, 1 − *y*, 1 − *z*. For **V**, there is a C—H⋯π inter­action between a hydrogen atom of the piperidine ring and the penta­fluoro­phenyl ring [*Cg*7⋯H4*A*^vii^ = 2.865 Å; *Cg*7 is the centroid of ring C14–C19; symmetry code: (vii) (x) −*x*, 1 − *y*, 1 − *z*]. This C—H⋯π inter­action is the shortest identified of the crystal structures presented. C—H⋯π inter­actions also exist between the two non-fluorinated phenyl rings of neighbouring mol­ecules [*Cg*8⋯H12^viii^ = 3.550 Å and *Cg*8⋯H11^viii^ = 3.748 Å; *Cg*8 is the centroid of ring C8–C13; symmetry code: (viii) −*x*, *y*, *z*] and between piperidine ring hydrogen atoms and non-fluorinated phenyl rings [*Cg*9⋯H1*B*^ix^ = 3.220 Å and *Cg*9⋯H3*B*^ix^ = 3.426 Å; *Cg*9 is the centroid of ring C8–C13; symmetry code: (ix) 1 − *x*, 1 − *y*, −*z*].

## 4. Database survey

A search of the Cambridge Structural Database (version 5.45, update in June 2024; Groom *et al.*; 2016 ▸) for phenidine deriv­atives resulted in four hits. All four hits are 2-methoxphenidine (**1b**) with a variety of solvates, some unknown (REBKOC; Jurásek *et al.*, 2022 ▸), and bromo- and chloro-zincate ions (REBLOD and REBLIX; Jurásek *et al.*, 2022 ▸). Entry FIDHIN (Jurásek *et al.*, 2023 ▸) is the hydro­chloride salt of the *R*-isomer of **1b** and as such is comparable to (**V**) due to the presence of a piperidine ring. Similar to (**V**), it has N—H⋯Cl distances of 2.120 and 2.123 Å (two mol­ecules in the asymmetric unit). The piperidine ring is the chair conformation, which is again directly comparable to (**V**). Entry REBKOC, mirrors that of FIDHIN except a chloro­form solvent mol­ecule is present in the asymmetric unit. It has an N—H—Cl distance of 2.209 Å and a Cl_3_C—H⋯*A* distance of 2.387 Å; the presence of this solvent mol­ecule has elongated the distance. The remaining two entries REBLOD and REBLIX (Jurásek *et al.*, 2022 ▸) both possess ZnCl_2_Br_4_^2−^ and ZnCl_2_Br_4_^2−^ ions in the asymmetric unit cell. Again, the piperidine ring is in the chair conformation for both REBLOD and REBLIX.

## 5. Synthesis and crystallization

**General method for di­aryl­ethyl­amine synthesis**

All diphenidine derivatives and analogues were synthesized using an adaptation of the published method (Le Gall *et al.*, 2009 ▸). The following modifications were applied to the published method: To zinc dust (2.0 g, 30 mmol) suspended in aceto­nitrile (40 mL), was added benzyl bromide (0.4 mL, 3.4 mmol) and trifluoro­acetic acid (0.2 mL). The resulting solution was stirred for 5 minutes and then benzyl bromide (3.0 mL, 25 mmol), the required amine (0.99 mL, 10 mmol) followed by the pre-requisite benzaldehyde (11 mmol), were introduced to the mixture, and the solution was stirred at room temperature for an additional 1 h. The resulting solution was poured into a saturated aqueous NH_4_Cl solution (150 mL) and extracted with di­chloro­methane (2 × 100 mL). The combined organic layers were dried (MgSO_4_) and concentrated *in vacuo* to give a crude yellowish oil. The oil was then dissolved in diethyl ether (150 mL) and concentrated sulfuric acid (0.75 mL) was added dropwise to the vigorously stirred solution. After five minutes, the precipitated ammonium salt was filtered, washed with diethyl ether (2 × 50 mL) and air dried for 5–10 minutes. The ammonium salt was re-dissolved in aqueous sodium hydroxide (5% *w*/*v*, 150 mL) and then extracted with di­chloro­methane (2 × 100 mL). The combined organic fractions were again dried (MgSO_4_) and concentrated *in vacuo* to give a yellow oil. The oil was dissolved in diethyl ether (200 mL), treated with hydrogen chloride (4 *M* in dioxane, 3.0 mL, 12 mmol) and left to stand for 5 minutes. The crystallized products were filtered and washed sequentially with the minimum amount of ice-cold acetone and if necessary an ice-cold mixture of ethyl acetate–diethyl ether (1:5) to afford the corresponding hydro­chloride salts as colourless to off-white powders.

(**I**) afforded 0.40 g (15%) of a white powder. Colourless crystals suitable for X-ray diffraction were grown from EtOAc/diethyl ether. ^1^H NMR (400 MHz, CD_2_Cl_2_): δ 7.4–7.5 (*m*, 1 H), 7.1–7.2 (*m*, 5 H), 7.0 (*br. s*, 1 H), 6.9 (*br. s*, 1 H), 4.9 (*dd*, *J* = 12.36, 2.75 Hz, 1 H), 4.0 (*dd*, *J* = 12.82, 3.66 Hz, 1 H), 3.6–3.7 (*m*, 1 H), 2.8 (*br. s*, 3 H), 2.7 (*br. s*., 3 H). ^13^C{^1^H} NMR (101 MHz, CD_2_Cl_2_): δ 162.5 (*dd*, *J* = 251.12, 7.67 Hz, C-F), 135.8, 133.6, 133.5, 133.4, 129.3, 129.1, 127.7, 113.5, 112.7, 107.0, 61.8, 43.0, 38.4, 34.7. ^19^F NMR (56 MHz, CD_2_Cl_2_): δ −111.21 (*br. s*, 2F). FT-IR (ATR, cm^−1^) 2306[RM1], 1624 (C=O), 1457 (C=C). M.p. = 385–387 K.

(**II**) afforded 2.24 g (64%) of a white powder. Colourless crystals suitable for X-ray diffraction were grown from DCM/diethyl ether. ^1^H NMR (400 MHz, DMSO-*d_6_*) δ 10.26 (*br. s*, 1H, NH), 9.18 (*br. s*, 1 H, NH), 7.02–7.20 (*m*, 7H, Ar-H), 7.50 (*dd*, 1H, *J* = 11.2, 4.2 Hz, Ar-H), 4.75 (*m*, 1H, NHCHCH_2_), 3.65 (*dd*, 1 H, *J* = 12.8, 4.8 Hz, NHCHCH_2_), 3.18 (*m*, 1 H, NHCHCH_2_), 2.95 (*m*, 2 H, NHCH_2_CH_3_), 1.28 (*t*, 3 H, *J* = 7.0 Hz, NHCH_2_CH_3_). ^19^F{^1^H} NMR (400 MHz, DMSO-*d*_6_) δ −113.68 (*s*, 2F); IR (ATR, cm^−1^): 2944 (C—H), 2670 (C—H), 1475 (C=C), 1202 (C—F). M.p. = 478 K.

(**III**) afforded 1.93 g (67%) of a white powder. Colourless crystals suitable for X-ray diffraction were grown from CHCl_3_/diethyl ether. ^1^H NMR (400 MHz, DMSO-*d_6_*) δ 10.3 (*d*, *J* = 9.16 Hz, 1 H), 8.9 (*dd*, *J* = 11.91, 5.04 Hz, 1 H), 7.4–7.5 (*m*, 1 H), 7.1–7.3 (*m*, 4 H), 7.0 (*dd*, *J* = 7.56, 2.06 Hz, 2 H), 6.9 (*s*, 1 H), 4.7 (*dd*, *J* = 11.68, 4.35 Hz, 1 H), 3.7 (*dd*, *J* = 12.82, 4.12 Hz, 1 H), 3.3–3.4 (*m*, 1 H), 1.4 (*s*, 9 H). ^13^C{^1^H} NMR (101 MHz, (CD_3_)_2_SO) δ 135.4, 132.2, 132.1, 132.0, 128.7, 128.2, 126.9, 112.5, 112.3, 111.8, 111.6, 111.4, 58.8, 49.4, 38.1, 25.3. ^19^F NMR (56 MHz, (CD_3_)_2_SO) δ −109.94 (*br. s*, 1F), −116.79 (*br. s*, 1F). FT-IR (ATR, cm^−1^) 2612, 1625, 1565, 1467; M.p. = 535–538 K.

(**IV**) afforded 2.22 g (77%) of a white powder. Colourless crystals suitable for X-ray diffraction were grown from DCM/diethyl ether. ^1^H NMR (400 MHz, CD_2_Cl_2_) δ 7.4 (*tt*, *J* = 8.47, 6.41 Hz, 1 H), 7.1–7.2 (*m*, 5 H), 7.0 (*br. s*., 1 H), 6.8 (*br. s*., 1 H), 4.9 (*dd*, *J* = 12.14, 3.89 Hz, 1 H), 3.9 (*dd*, *J* = 13.28, 4.58 Hz, 2 H), 3.6 (*t*, *J* = 12.59 Hz, 2 H), 2.9 (*br. s*., 1 H), 2.8 (*br. s*., 1 H), 2.2 (*br. s*., 5 H), 1.9 (*br. s*., 2 H). ^13^C{^1^H} NMR (101 MHz, CD_2_Cl_2_) δ 161.6 (*dd*, *J* = 250.16, 7.67 Hz) C—F, 136.0, 133.3, 133.2, 133.1, 129.2, 129.1, 127.6, 113.4, 112.5, 109.0, 108.8, 108.7, 59.9, 50.6, 35.9, 23.4, 23.2. ^19^F NMR (56 MHz, CD_2_Cl_2_) δ −108.10 (*br. s*, 1F), −114.18 (*br. s*, 1F). FT-IR (ATR, cm^−1^) 2352, 1623, 1460. M.p. = 486–488 K.

(**V**) afforded 1.55 g (52%) of a white powder. Colourless crystals suitable for X-ray diffraction were grown from CHCl_3_/diethyl ether. ^1^H NMR (400 MHz, CD_2_Cl_2_) δ 7.11–7.28 (*m*, 5 H), 4.90 (*d*, *J* = 12.36 Hz, 1 H), 4.23 (*dd*, *J* = 12.82, 3.66 Hz, 1 H), 3.80 (*d*, *J* = 11.45 Hz, 1 H), 3.44–3.55 (*m*, 2 H), 2.57–2.70 (*m*, 1 H), 2.48 (*d*, *J* = 9.62 Hz, 1 H), 2.24–2.38 (*m*, 1 H), 1.91 (*t*, *J* = 14.88 Hz, 2 H), 1.80 (*d*, *J* = 13.74 Hz, 1 H), 1.22–1.37 (*m*, 1 H). ^13^C{^1^H} NMR (101 MHz, CD_2_Cl_2_) δ 120.9, 115.0, 114.6, 113.5, 91.0, 48.2, 34.5, 19.2, 9.0, 8.9, 8.1. ^19^F NMR (56 MHz, CD_2_Cl_2_) δ −134.86, −139.28, −152.81 (*t*, *J* = 22.4 Hz), −162.69. FT-IR (ATR, cm^−1^) 2309, 1503, 1459. M.p. = 502–504 K.

## 6. Refinement

Crystal data, data collection and structure refinement details are summarized in Table 6 ▸. Non-hydrogen atoms were refined anisotropically. Hydrogen atoms were included as riding contributions in idealized positions with isotropic displacement parameters *U*_iso_(H) = 1.2*U*_eq_(C) (1.5 for methyl groups). All structures were solved by direct methods. For (**II**) the terminal carbon of the ethyl group (C15, C15*A*), is disordered over two positions [0.707 (5):0.293 (5) occupancy]. All non H-atoms were refined anisotropically. The H atoms were placed in calculated positions, except for H1*N*1 (**I**), H1*A* and H1*B* (**II**), H1′ and H2 (**IV**) and H2 (**V**), which were all found. For (**V**), a DFIX instruction was applied to N1—H1′ and N2—H2 (fixed at 0.98 Å).

## Supplementary Material

Crystal structure: contains datablock(s) V, I, IV, II, III, global. DOI: 10.1107/S2056989025001288/ee2011sup1.cif

Structure factors: contains datablock(s) V. DOI: 10.1107/S2056989025001288/ee2011Vsup2.hkl

Structure factors: contains datablock(s) I. DOI: 10.1107/S2056989025001288/ee2011Isup3.hkl

Structure factors: contains datablock(s) IV. DOI: 10.1107/S2056989025001288/ee2011IVsup4.hkl

Structure factors: contains datablock(s) II. DOI: 10.1107/S2056989025001288/ee2011IIsup5.hkl

Structure factors: contains datablock(s) III. DOI: 10.1107/S2056989025001288/ee2011IIIsup6.hkl

CCDC references: 2423059, 2423058, 2423057, 2423056, 2423055

Additional supporting information:  crystallographic information; 3D view; checkCIF report

## Figures and Tables

**Table 1:** Hydrogen-bond geometry (Å, °) for (**I**)

*D*—H⋯*A*	*D*—H	H⋯*A*	*D*⋯*A*	*D*—H⋯*A*
N1—H1*N*1⋯Cl1	0.93 (2)	2.08 (2)	3.0006 (17)	167.3 (19)

**Table 2:** Hydrogen-bond geometry (Å, °) for (**II**)

*D*—H⋯*A*	*D*—H	H⋯*A*	*D*⋯*A*	*D*—H⋯*A*
N1—H1*B*⋯Cl1	0.89 (2)	2.30 (2)	3.1417 (14)	156.0 (16)

**Table 3:** Hydrogen-bond geometry (Å, °) for (**III**)

*D*—H⋯*A*	*D*—H	H⋯*A*	*D*⋯*A*	*D*—H⋯*A*
N1—H1*A*⋯Cl1	0.92 (2)	2.21 (3)	3.115 (2)	167.7 (19)
N1—H1*B*⋯Cl1^i^	0.95 (2)	2.31 (2)	3.1684 (19)	151 (2)

Symmetry code: (i) 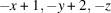.

**Table 4:** Hydrogen-bond geometry (Å, °) for (**IV**)

*D*—H⋯*A*	*D*—H	H⋯*A*	*D*⋯*A*	*D*—H⋯*A*
N1—H1′⋯Cl1	0.99 (1)	2.07 (2)	3.021 (5)	163 (6)
N2—H2⋯Cl2	0.98 (1)	2.08 (2)	3.052 (5)	169 (6)

**Table 5:** Hydrogen-bond geometry (Å, °) for (**V**)

*D*—H⋯*A*	*D*—H	H⋯*A*	*D*⋯*A*	*D*—H⋯*A*
N1—H1⋯Cl1	0.85 (3)	2.20 (3)	3.051 (3)	178 (3)

**Table 6:** Experimental details

	(**I**)	(**II**)	(**III**)	(**IV**)	(**v**)
Crystal data					
Chemical formula	C_16_H_18_F_2_N^+^·Cl^−^	2C_16_H_18_F_2_N^+^·2Cl^−^·CH_2_Cl_2_	C_18_H_22_F_2_N^+^·Cl^−^	C_18_H_20_F_2_N^+^·Cl^−^	C_19_H_19_F_5_N^+^·Cl^−^
*M*_r_	297.76	680.45	325.81	323.80	391.80
Crystal system, space group	Monoclinic, *P*2_1_/*c*	Monoclinic, *I*2/*a*	Monoclinic, *P*2_1_/*c*	Triclinic, *P* 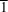	Monoclinic, *P*2_1_/*c*
Temperature (K)	123	123	123	123	123
*a*, *b*, *c* (Å)	7.9474 (3), 12.7652 (5), 15.3998 (7)	22.9963 (14), 7.8729 (5), 19.033 (1)	11.3115 (6), 10.5400 (5), 14.8039 (7)	8.1365 (4), 12.7421 (10), 16.0451 (8)	9.3155 (5), 22.2529 (13), 8.2699 (3)
α, β, γ (°)	90, 99.368 (4), 90	90, 92.130 (5), 90	90, 105.044 (5), 90	88.059 (5), 82.349 (4), 86.140 (5)	90, 90.165 (5), 90
*V* (Å^3^)	1541.48 (11)	3443.5 (4)	1704.48 (15)	1644.42 (17)	1714.32 (15)
*Z*	4	4	4	4	4
Radiation type	Mo *K*α	Mo *K*α	Mo *K*α	Mo *K*α	Mo *K*α
μ (mm^−1^)	0.26	0.39	0.24	0.25	0.28
Crystal size (mm)	0.5 × 0.4 × 0.2	0.3 × 0.2 × 0.1	0.4 × 0.2 × 0.1	0.5 × 0.4 × 0.3	0.2 × 0.1 × 0.05

Data collection					
Diffractometer	Oxford Diffraction Xcalibur	Oxford Diffraction Xcalibur	Oxford Diffraction Xcalibur	Oxford Diffraction Xcalibur	Oxford Diffraction Xcalibur
Absorption correction	Analytical (*CrysAlis PRO*; Agilent 2014 ▸)	Analytical (*SADABS*; Krause *et al.*, 2015 ▸)	Analytical (*SADABS*; Krause *et al.*, 2015 ▸)	Analytical (*SADABS*; Krause *et al.*, 2015 ▸)	Analytical (*SADABS*; Krause *et al.*, 2015 ▸)
*T*_min_, *T*_max_	0.884, 0.950	0.911, 0.962	0.944, 0.976	0.888, 0.928	0.967, 0.986
No. of measured, independent and observed [*I* > 2σ(*I*)] reflections	6424, 2715, 2305	12738, 3034, 2813	7223, 3008, 2343	13277, 5775, 4892	5523, 2896, 2117
*R*_int_	0.027	0.021	0.036	0.032	0.049
(sin θ/λ)_max_ (Å^−1^)	0.595	0.595	0.595	0.595	0.595

Refinement					
*R*[*F*^2^ > 2σ(*F*^2^)], *wR*(*F*^2^), *S*	0.040, 0.088, 1.06	0.031, 0.077, 1.07	0.049, 0.095, 1.06	0.090, 0.206, 1.19	0.056, 0.123, 1.01
No. of reflections	2715	3034	3008	5775	2896
No. of parameters	186	224	210	403	239
No. of restraints	1	0	0	2	0
H-atom treatment	H atoms treated by a mixture of independent and constrained refinement	H atoms treated by a mixture of independent and constrained refinement	H atoms treated by a mixture of independent and constrained refinement	H atoms treated by a mixture of independent and constrained refinement	H atoms treated by a mixture of independent and constrained refinement
Δρ_max_, Δρ_min_ (e Å^−3^)	0.20, −0.26	0.25, −0.22	0.22, −0.25	0.59, −0.34	0.30, −0.25

Computer programs: *CrysAlis PRO* (Agilent, 2014 ▸), *SHELXS97* (Sheldrick, 2008 ▸), *SHELXT2014/5* (Sheldrick, 2015*a* ▸), *SHELXL2018/3* (Sheldrick, 2015*b* ▸) and *X-SEED* (Barbour, 2020 ▸).

## supplementary crystallographic information

### 1-[1-(2,3,4,5,6-Pentafluorophenyl)-2-phenylethyl]piperidin-1-ium chloride (V) Crystal data

**Table:**

C_19_H_19_F_5_N^+^·Cl^−^	*F*(000) = 808
*M**_r_* = 391.80	*D*_x_ = 1.518 Mg m^−^^3^
Monoclinic, *P*2_1_/*c*	Mo *K*α radiation, λ = 0.71073 Å
*a* = 9.3155 (5) Å	Cell parameters from 9843 reflections
*b* = 22.2529 (13) Å	θ = 3.0–26.5°
*c* = 8.2699 (3) Å	µ = 0.28 mm^−^^1^
β = 90.165 (5)°	*T* = 123 K
*V* = 1714.32 (15) Å^3^	Block, colourless
*Z* = 4	0.2 × 0.1 × 0.05 mm

### 1-[1-(2,3,4,5,6-Pentafluorophenyl)-2-phenylethyl]piperidin-1-ium chloride (V) Data collection

**Table:**

Oxford Diffraction Xcalibur diffractometer	2117 reflections with *I* > 2σ(*I*)
Radiation source: fine-focus sealed tube	*R*_int_ = 0.049
scans in phi and ω	θ_max_ = 25.0°, θ_min_ = 3.1°
Absorption correction: analytical (SADABS; Krause *et al.*, 2015)	*h* = −10→11
*T*_min_ = 0.967, *T*_max_ = 0.986	*k* = −18→26
5523 measured reflections	*l* = −8→9
2896 independent reflections	

### 1-[1-(2,3,4,5,6-Pentafluorophenyl)-2-phenylethyl]piperidin-1-ium chloride (V) Refinement

**Table:**

Refinement on *F*^2^	0 restraints
Least-squares matrix: full	Hydrogen site location: mixed
*R*[*F*^2^ > 2σ(*F*^2^)] = 0.056	H atoms treated by a mixture of independent and constrained refinement
*wR*(*F*^2^) = 0.123	*w* = 1/[σ^2^(*F*_o_^2^) + (0.0452*P*)^2^ + 0.3121*P*] where *P* = (*F*_o_^2^ + 2*F*_c_^2^)/3
*S* = 1.01	(Δ/σ)_max_ = 0.001
2896 reflections	Δρ_max_ = 0.30 e Å^−^^3^
239 parameters	Δρ_min_ = −0.25 e Å^−^^3^

### 1-[1-(2,3,4,5,6-Pentafluorophenyl)-2-phenylethyl]piperidin-1-ium chloride (V) Special details

**Table:**

Geometry. All esds (except the esd in the dihedral angle between two l.s. planes) are estimated using the full covariance matrix. The cell esds are taken into account individually in the estimation of esds in distances, angles and torsion angles; correlations between esds in cell parameters are only used when they are defined by crystal symmetry. An approximate (isotropic) treatment of cell esds is used for estimating esds involving l.s. planes.

### 1-[1-(2,3,4,5,6-Pentafluorophenyl)-2-phenylethyl]piperidin-1-ium chloride (V) Fractional atomic coordinates and isotropic or equivalent isotropic displacement parameters (Å^2^)

**Table:**

	*x*	*y*	*z*	*U*_iso_*/*U*_eq_	
Cl1	0.24269 (9)	0.51019 (4)	0.68922 (9)	0.0315 (2)	
F1	0.07331 (19)	0.42667 (8)	0.0555 (2)	0.0327 (5)	
F5	0.31205 (19)	0.36912 (8)	0.53597 (19)	0.0307 (5)	
F4	0.1190 (2)	0.28212 (8)	0.5801 (2)	0.0404 (5)	
F2	−0.1166 (2)	0.33964 (10)	0.1010 (2)	0.0474 (6)	
F3	−0.0977 (2)	0.26613 (9)	0.3625 (2)	0.0492 (6)	
C14	0.1989 (3)	0.40279 (14)	0.2969 (3)	0.0218 (7)	
C18	0.1081 (4)	0.31807 (14)	0.4497 (4)	0.0281 (8)	
C6	0.3167 (3)	0.44932 (13)	0.2723 (3)	0.0215 (7)	
H6	0.395538	0.438117	0.348882	0.026*	
C5	0.1334 (3)	0.53469 (14)	0.2445 (4)	0.0256 (8)	
H5A	0.055024	0.505280	0.260780	0.031*	
H5B	0.148162	0.539641	0.126760	0.031*	
C4	0.0922 (3)	0.59416 (15)	0.3182 (4)	0.0308 (8)	
H4A	0.070518	0.588238	0.434277	0.037*	
H4B	0.003969	0.609188	0.264765	0.037*	
C8	0.4205 (3)	0.38395 (14)	0.0529 (3)	0.0246 (8)	
C1	0.3883 (3)	0.55697 (14)	0.3002 (4)	0.0267 (8)	
H1A	0.477032	0.541589	0.351631	0.032*	
H1B	0.407412	0.562601	0.183476	0.032*	
C19	0.2066 (3)	0.36364 (14)	0.4273 (3)	0.0229 (7)	
C10	0.5654 (4)	0.29493 (15)	0.0849 (4)	0.0320 (8)	
H10	0.641115	0.274303	0.138504	0.038*	
C16	−0.0118 (4)	0.34743 (16)	0.2098 (4)	0.0310 (8)	
C2	0.3486 (3)	0.61639 (15)	0.3748 (4)	0.0286 (8)	
H2A	0.427250	0.645549	0.357103	0.034*	
H2B	0.336184	0.611273	0.492819	0.034*	
N1	0.2689 (3)	0.51174 (11)	0.3216 (3)	0.0208 (6)	
C7	0.3833 (3)	0.44745 (14)	0.1024 (3)	0.0258 (8)	
H7A	0.314639	0.464662	0.023408	0.031*	
H7B	0.471239	0.472407	0.101079	0.031*	
C15	0.0873 (3)	0.39271 (14)	0.1886 (3)	0.0242 (8)	
C17	−0.0007 (4)	0.30990 (15)	0.3412 (4)	0.0323 (9)	
C12	0.3791 (4)	0.29713 (16)	−0.1148 (4)	0.0327 (9)	
H12	0.327115	0.277949	−0.199197	0.039*	
C3	0.2103 (4)	0.64091 (15)	0.3016 (4)	0.0317 (8)	
H3A	0.182086	0.678299	0.358033	0.038*	
H3B	0.225342	0.650474	0.185948	0.038*	
C11	0.4883 (4)	0.26676 (15)	−0.0364 (4)	0.0315 (9)	
H11	0.510387	0.226548	−0.065800	0.038*	
C9	0.5318 (3)	0.35331 (15)	0.1277 (4)	0.0279 (8)	
H9	0.586002	0.372763	0.209846	0.033*	
C13	0.3452 (4)	0.35548 (15)	−0.0706 (4)	0.0303 (8)	
H13	0.270025	0.376141	−0.125034	0.036*	
H1	0.261 (4)	0.5105 (14)	0.425 (4)	0.038 (10)*	

### 1-[1-(2,3,4,5,6-Pentafluorophenyl)-2-phenylethyl]piperidin-1-ium chloride (V) Atomic displacement parameters (Å^2^)

**Table:**

	*U*^11^	*U*^22^	*U*^33^	*U*^12^	*U*^13^	*U*^23^
Cl1	0.0238 (5)	0.0421 (5)	0.0286 (4)	0.0016 (4)	0.0007 (4)	0.0024 (4)
F1	0.0234 (11)	0.0457 (12)	0.0289 (9)	−0.0008 (10)	−0.0060 (8)	0.0025 (8)
F5	0.0222 (11)	0.0387 (12)	0.0313 (9)	−0.0010 (9)	−0.0049 (8)	0.0063 (8)
F4	0.0404 (13)	0.0362 (12)	0.0446 (11)	−0.0041 (10)	0.0081 (10)	0.0109 (9)
F2	0.0290 (12)	0.0668 (15)	0.0464 (11)	−0.0172 (11)	−0.0084 (10)	−0.0126 (10)
F3	0.0418 (14)	0.0461 (13)	0.0596 (13)	−0.0280 (12)	0.0088 (11)	−0.0072 (10)
C14	0.0128 (17)	0.0257 (17)	0.0271 (16)	0.0031 (15)	0.0054 (14)	−0.0046 (13)
C18	0.030 (2)	0.0277 (19)	0.0271 (17)	0.0013 (17)	0.0061 (16)	0.0021 (14)
C6	0.0123 (17)	0.0248 (18)	0.0273 (16)	0.0005 (15)	0.0013 (14)	−0.0005 (13)
C5	0.0143 (18)	0.034 (2)	0.0285 (16)	0.0029 (16)	−0.0038 (14)	−0.0015 (14)
C4	0.0173 (19)	0.039 (2)	0.0362 (18)	0.0073 (17)	−0.0046 (16)	−0.0018 (15)
C8	0.0188 (18)	0.0282 (18)	0.0268 (16)	−0.0001 (16)	0.0079 (15)	0.0034 (14)
C1	0.0140 (18)	0.033 (2)	0.0329 (18)	−0.0044 (16)	0.0020 (15)	0.0030 (14)
C19	0.0171 (18)	0.0295 (19)	0.0222 (16)	0.0020 (16)	0.0003 (14)	−0.0027 (14)
C10	0.027 (2)	0.037 (2)	0.0325 (18)	0.0074 (18)	0.0072 (16)	0.0058 (16)
C16	0.0186 (19)	0.044 (2)	0.0302 (18)	−0.0056 (18)	0.0010 (16)	−0.0122 (16)
C2	0.0187 (19)	0.0299 (19)	0.0373 (18)	−0.0037 (16)	0.0022 (15)	0.0005 (15)
N1	0.0128 (15)	0.0278 (15)	0.0220 (14)	−0.0014 (13)	−0.0002 (12)	0.0023 (12)
C7	0.0196 (19)	0.0291 (19)	0.0288 (17)	0.0010 (16)	0.0056 (15)	0.0031 (14)
C15	0.0213 (19)	0.0306 (19)	0.0207 (16)	0.0004 (16)	0.0025 (14)	−0.0021 (14)
C17	0.025 (2)	0.0299 (19)	0.042 (2)	−0.0084 (17)	0.0106 (17)	−0.0100 (16)
C12	0.025 (2)	0.043 (2)	0.0299 (18)	−0.0082 (19)	0.0031 (16)	−0.0075 (16)
C3	0.030 (2)	0.0292 (19)	0.0355 (18)	0.0026 (17)	0.0013 (17)	0.0020 (15)
C11	0.035 (2)	0.0279 (19)	0.0321 (19)	0.0015 (18)	0.0110 (17)	−0.0003 (15)
C9	0.0194 (19)	0.035 (2)	0.0289 (17)	−0.0028 (16)	0.0026 (15)	−0.0007 (14)
C13	0.0197 (19)	0.043 (2)	0.0284 (17)	0.0030 (17)	0.0048 (15)	0.0011 (15)

### 1-[1-(2,3,4,5,6-Pentafluorophenyl)-2-phenylethyl]piperidin-1-ium chloride (V) Geometric parameters (Å, º)

**Table:**

F1—C15	1.342 (3)	C1—C2	1.506 (4)
F5—C19	1.335 (3)	C1—N1	1.511 (4)
F4—C18	1.347 (3)	C1—H1A	0.9900
F2—C16	1.338 (4)	C1—H1B	0.9900
F3—C17	1.340 (4)	C10—C9	1.383 (5)
C14—C15	1.388 (4)	C10—C11	1.382 (5)
C14—C19	1.388 (4)	C10—H10	0.9500
C14—C6	1.523 (4)	C16—C17	1.374 (5)
C18—C17	1.364 (5)	C16—C15	1.378 (4)
C18—C19	1.380 (4)	C2—C3	1.523 (4)
C6—N1	1.515 (4)	C2—H2A	0.9900
C6—C7	1.538 (4)	C2—H2B	0.9900
C6—H6	1.0000	N1—H1	0.85 (3)
C5—N1	1.502 (4)	C7—H7A	0.9900
C5—C4	1.507 (4)	C7—H7B	0.9900
C5—H5A	0.9900	C12—C11	1.381 (5)
C5—H5B	0.9900	C12—C13	1.386 (5)
C4—C3	1.521 (4)	C12—H12	0.9500
C4—H4A	0.9900	C3—H3A	0.9900
C4—H4B	0.9900	C3—H3B	0.9900
C8—C9	1.385 (4)	C11—H11	0.9500
C8—C13	1.390 (4)	C9—H9	0.9500
C8—C7	1.512 (4)	C13—H13	0.9500
			
C15—C14—C19	115.9 (3)	C1—C2—C3	111.1 (3)
C15—C14—C6	124.2 (3)	C1—C2—H2A	109.4
C19—C14—C6	119.6 (3)	C3—C2—H2A	109.4
F4—C18—C17	120.1 (3)	C1—C2—H2B	109.4
F4—C18—C19	119.7 (3)	C3—C2—H2B	109.4
C17—C18—C19	120.2 (3)	H2A—C2—H2B	108.0
N1—C6—C14	112.0 (2)	C5—N1—C1	110.0 (2)
N1—C6—C7	113.0 (2)	C5—N1—C6	116.4 (2)
C14—C6—C7	113.3 (3)	C1—N1—C6	111.2 (2)
N1—C6—H6	105.9	C5—N1—H1	111 (2)
C14—C6—H6	105.9	C1—N1—H1	102 (2)
C7—C6—H6	105.9	C6—N1—H1	105 (2)
N1—C5—C4	110.0 (3)	C8—C7—C6	111.5 (2)
N1—C5—H5A	109.7	C8—C7—H7A	109.3
C4—C5—H5A	109.7	C6—C7—H7A	109.3
N1—C5—H5B	109.7	C8—C7—H7B	109.3
C4—C5—H5B	109.7	C6—C7—H7B	109.3
H5A—C5—H5B	108.2	H7A—C7—H7B	108.0
C5—C4—C3	112.2 (3)	F1—C15—C16	116.9 (3)
C5—C4—H4A	109.2	F1—C15—C14	120.6 (3)
C3—C4—H4A	109.2	C16—C15—C14	122.5 (3)
C5—C4—H4B	109.2	F3—C17—C18	120.7 (3)
C3—C4—H4B	109.2	F3—C17—C16	119.8 (3)
H4A—C4—H4B	107.9	C18—C17—C16	119.5 (3)
C9—C8—C13	118.6 (3)	C11—C12—C13	120.2 (3)
C9—C8—C7	120.7 (3)	C11—C12—H12	119.9
C13—C8—C7	120.6 (3)	C13—C12—H12	119.9
C2—C1—N1	110.8 (2)	C4—C3—C2	109.3 (3)
C2—C1—H1A	109.5	C4—C3—H3A	109.8
N1—C1—H1A	109.5	C2—C3—H3A	109.8
C2—C1—H1B	109.5	C4—C3—H3B	109.8
N1—C1—H1B	109.5	C2—C3—H3B	109.8
H1A—C1—H1B	108.1	H3A—C3—H3B	108.3
F5—C19—C18	117.7 (3)	C12—C11—C10	119.9 (3)
F5—C19—C14	120.2 (3)	C12—C11—H11	120.0
C18—C19—C14	122.1 (3)	C10—C11—H11	120.0
C9—C10—C11	119.6 (3)	C10—C9—C8	121.2 (3)
C9—C10—H10	120.2	C10—C9—H9	119.4
C11—C10—H10	120.2	C8—C9—H9	119.4
F2—C16—C17	120.4 (3)	C12—C13—C8	120.4 (3)
F2—C16—C15	119.8 (3)	C12—C13—H13	119.8
C17—C16—C15	119.7 (3)	C8—C13—H13	119.8

### 1-[1-(2,3,4,5,6-Pentafluorophenyl)-2-phenylethyl]piperidin-1-ium chloride (V) Hydrogen-bond geometry (Å, º)

**Table:**

*D*—H···*A*	*D*—H	H···*A*	*D*···*A*	*D*—H···*A*
N1—H1···Cl1	0.85 (3)	2.20 (3)	3.051 (3)	178 (3)

### [1-(2,6-Difluorophenyl)-2-phenylethyl]dimethylazanium chloride (I) Crystal data

**Table:**

C_16_H_18_F_2_N^+^·Cl^−^	*F*(000) = 624
*M**_r_* = 297.76	*D*_x_ = 1.283 Mg m^−^^3^
Monoclinic, *P*2_1_/*c*	Mo *K*α radiation, λ = 0.71073 Å
*a* = 7.9474 (3) Å	Cell parameters from 8632 reflections
*b* = 12.7652 (5) Å	θ = 3.0–26.6°
*c* = 15.3998 (7) Å	µ = 0.26 mm^−^^1^
β = 99.368 (4)°	*T* = 123 K
*V* = 1541.48 (11) Å^3^	Block, colourless
*Z* = 4	0.5 × 0.4 × 0.2 mm

### [1-(2,6-Difluorophenyl)-2-phenylethyl]dimethylazanium chloride (I) Data collection

**Table:**

Oxford Diffraction Xcalibur diffractometer	2305 reflections with *I* > 2σ(*I*)
Radiation source: fine-focus sealed tube	*R*_int_ = 0.027
scans in phi and ω	θ_max_ = 25.0°, θ_min_ = 3.1°
Absorption correction: analytical (CrysAlisPro; Agilent 2014)	*h* = −9→9
*T*_min_ = 0.884, *T*_max_ = 0.950	*k* = −14→15
6424 measured reflections	*l* = −18→16
2715 independent reflections	

### [1-(2,6-Difluorophenyl)-2-phenylethyl]dimethylazanium chloride (I) Refinement

**Table:**

Refinement on *F*^2^	1 restraint
Least-squares matrix: full	Hydrogen site location: mixed
*R*[*F*^2^ > 2σ(*F*^2^)] = 0.040	H atoms treated by a mixture of independent and constrained refinement
*wR*(*F*^2^) = 0.088	*w* = 1/[σ^2^(*F*_o_^2^) + (0.0281*P*)^2^ + 0.7459*P*] where *P* = (*F*_o_^2^ + 2*F*_c_^2^)/3
*S* = 1.06	(Δ/σ)_max_ < 0.001
2715 reflections	Δρ_max_ = 0.20 e Å^−^^3^
186 parameters	Δρ_min_ = −0.26 e Å^−^^3^

### [1-(2,6-Difluorophenyl)-2-phenylethyl]dimethylazanium chloride (I) Special details

**Table:**

Geometry. All esds (except the esd in the dihedral angle between two l.s. planes) are estimated using the full covariance matrix. The cell esds are taken into account individually in the estimation of esds in distances, angles and torsion angles; correlations between esds in cell parameters are only used when they are defined by crystal symmetry. An approximate (isotropic) treatment of cell esds is used for estimating esds involving l.s. planes.

### [1-(2,6-Difluorophenyl)-2-phenylethyl]dimethylazanium chloride (I) Fractional atomic coordinates and isotropic or equivalent isotropic displacement parameters (Å^2^)

**Table:**

	*x*	*y*	*z*	*U*_iso_*/*U*_eq_	
C8	0.8942 (2)	0.41635 (15)	0.29692 (12)	0.0230 (4)	
H8A	0.997147	0.441707	0.336303	0.028*	
H8B	0.918077	0.345200	0.276392	0.028*	
C3	0.4368 (2)	0.45234 (14)	0.28669 (13)	0.0235 (4)	
C7	0.5501 (2)	0.30531 (14)	0.22760 (13)	0.0234 (4)	
C2	0.5752 (2)	0.38564 (13)	0.28907 (12)	0.0188 (4)	
C14	0.8167 (2)	0.45121 (15)	0.13360 (13)	0.0286 (5)	
H14	0.825573	0.378177	0.123370	0.034*	
C6	0.4032 (2)	0.29047 (16)	0.16854 (13)	0.0295 (5)	
H6	0.392555	0.233545	0.128282	0.035*	
C10	0.8393 (2)	0.59657 (15)	0.23204 (13)	0.0251 (4)	
H10	0.863656	0.624451	0.289921	0.030*	
C5	0.2715 (2)	0.36051 (17)	0.16928 (14)	0.0346 (5)	
H5	0.168621	0.351866	0.128876	0.042*	
C12	0.7556 (3)	0.62399 (16)	0.07667 (14)	0.0357 (5)	
H12	0.722285	0.669788	0.028305	0.043*	
C11	0.7913 (3)	0.66314 (15)	0.16121 (14)	0.0303 (5)	
H11	0.782997	0.736312	0.170946	0.036*	
C4	0.2871 (2)	0.44303 (17)	0.22793 (14)	0.0318 (5)	
H4	0.197122	0.492047	0.227789	0.038*	
C13	0.7688 (3)	0.51778 (16)	0.06310 (14)	0.0384 (5)	
H13	0.744851	0.490217	0.005099	0.046*	
F1	0.45166 (14)	0.53325 (8)	0.34416 (8)	0.0334 (3)	
F2	0.68097 (14)	0.23779 (8)	0.22353 (8)	0.0329 (3)	
C1	0.7428 (2)	0.41269 (14)	0.34703 (11)	0.0186 (4)	
H1	0.729073	0.485786	0.368148	0.022*	
C9	0.8521 (2)	0.48917 (14)	0.21896 (12)	0.0204 (4)	
Cl1	1.14563 (7)	0.36912 (4)	0.51711 (4)	0.03725 (17)	
N1	0.7819 (2)	0.34594 (12)	0.42918 (10)	0.0248 (4)	
C15	0.6669 (3)	0.37192 (17)	0.49322 (14)	0.0363 (5)	
H15A	0.703925	0.333884	0.548299	0.055*	
H15B	0.549887	0.351557	0.468782	0.055*	
H15C	0.671182	0.447444	0.504801	0.055*	
C16	0.7872 (3)	0.23087 (15)	0.41449 (14)	0.0336 (5)	
H16A	0.672229	0.205541	0.391314	0.050*	
H16B	0.829292	0.195665	0.470357	0.050*	
H16C	0.863511	0.215610	0.372127	0.050*	
H1N1	0.893 (3)	0.3638 (16)	0.4538 (15)	0.039 (6)*	

### [1-(2,6-Difluorophenyl)-2-phenylethyl]dimethylazanium chloride (I) Atomic displacement parameters (Å^2^)

**Table:**

	*U*^11^	*U*^22^	*U*^33^	*U*^12^	*U*^13^	*U*^23^
C8	0.0196 (9)	0.0288 (10)	0.0196 (10)	−0.0001 (8)	−0.0003 (8)	−0.0001 (8)
C3	0.0274 (10)	0.0239 (10)	0.0210 (10)	−0.0010 (8)	0.0090 (8)	0.0012 (8)
C7	0.0241 (10)	0.0230 (9)	0.0234 (10)	−0.0028 (8)	0.0048 (8)	−0.0003 (8)
C2	0.0198 (9)	0.0210 (9)	0.0156 (9)	−0.0032 (7)	0.0033 (7)	0.0019 (8)
C14	0.0388 (12)	0.0233 (10)	0.0241 (11)	−0.0015 (9)	0.0064 (9)	−0.0025 (9)
C6	0.0299 (11)	0.0352 (11)	0.0219 (11)	−0.0117 (9)	−0.0004 (9)	−0.0028 (9)
C10	0.0257 (10)	0.0296 (10)	0.0205 (11)	−0.0053 (8)	0.0049 (8)	−0.0032 (9)
C5	0.0228 (10)	0.0516 (14)	0.0274 (12)	−0.0124 (10)	−0.0020 (9)	0.0082 (11)
C12	0.0502 (13)	0.0307 (11)	0.0262 (12)	−0.0007 (10)	0.0061 (10)	0.0109 (10)
C11	0.0386 (12)	0.0222 (10)	0.0317 (12)	−0.0025 (9)	0.0105 (9)	−0.0004 (9)
C4	0.0192 (10)	0.0413 (12)	0.0353 (13)	0.0027 (9)	0.0052 (9)	0.0120 (10)
C13	0.0638 (15)	0.0343 (12)	0.0167 (11)	−0.0026 (11)	0.0051 (10)	0.0008 (9)
F1	0.0348 (6)	0.0300 (6)	0.0367 (7)	0.0077 (5)	0.0094 (5)	−0.0057 (5)
F2	0.0351 (6)	0.0283 (6)	0.0339 (7)	0.0039 (5)	0.0018 (5)	−0.0116 (5)
C1	0.0219 (9)	0.0192 (9)	0.0141 (9)	0.0008 (7)	0.0010 (7)	0.0006 (8)
C9	0.0145 (9)	0.0260 (10)	0.0210 (10)	−0.0017 (7)	0.0042 (7)	0.0026 (8)
Cl1	0.0431 (3)	0.0269 (3)	0.0332 (3)	0.0104 (2)	−0.0191 (2)	−0.0082 (2)
N1	0.0298 (9)	0.0277 (9)	0.0159 (8)	−0.0003 (7)	0.0008 (7)	0.0024 (7)
C15	0.0497 (13)	0.0419 (12)	0.0202 (11)	0.0030 (10)	0.0141 (10)	0.0049 (10)
C16	0.0461 (13)	0.0251 (10)	0.0282 (12)	0.0029 (9)	0.0019 (10)	0.0076 (9)

### [1-(2,6-Difluorophenyl)-2-phenylethyl]dimethylazanium chloride (I) Geometric parameters (Å, º)

**Table:**

C8—C9	1.512 (3)	C5—C4	1.380 (3)
C8—C1	1.532 (3)	C5—H5	0.9500
C8—H8A	0.9900	C12—C13	1.378 (3)
C8—H8B	0.9900	C12—C11	1.380 (3)
C3—F1	1.353 (2)	C12—H12	0.9500
C3—C4	1.378 (3)	C11—H11	0.9500
C3—C2	1.386 (3)	C4—H4	0.9500
C7—F2	1.360 (2)	C13—H13	0.9500
C7—C6	1.371 (3)	C1—N1	1.515 (2)
C7—C2	1.388 (3)	C1—H1	1.0000
C2—C1	1.518 (2)	N1—C15	1.487 (3)
C14—C13	1.382 (3)	N1—C16	1.488 (2)
C14—C9	1.386 (3)	N1—H1N1	0.93 (2)
C14—H14	0.9500	C15—H15A	0.9800
C6—C5	1.378 (3)	C15—H15B	0.9800
C6—H6	0.9500	C15—H15C	0.9800
C10—C11	1.386 (3)	C16—H16A	0.9800
C10—C9	1.392 (3)	C16—H16B	0.9800
C10—H10	0.9500	C16—H16C	0.9800
			
C9—C8—C1	109.13 (14)	C3—C4—C5	118.14 (19)
C9—C8—H8A	109.9	C3—C4—H4	120.9
C1—C8—H8A	109.9	C5—C4—H4	120.9
C9—C8—H8B	109.9	C12—C13—C14	120.2 (2)
C1—C8—H8B	109.9	C12—C13—H13	119.9
H8A—C8—H8B	108.3	C14—C13—H13	119.9
F1—C3—C4	118.01 (17)	N1—C1—C2	113.84 (14)
F1—C3—C2	117.86 (16)	N1—C1—C8	111.51 (14)
C4—C3—C2	124.11 (18)	C2—C1—C8	113.39 (15)
F2—C7—C6	117.17 (17)	N1—C1—H1	105.8
F2—C7—C2	118.28 (16)	C2—C1—H1	105.8
C6—C7—C2	124.53 (18)	C8—C1—H1	105.8
C3—C2—C7	114.24 (16)	C14—C9—C10	118.25 (17)
C3—C2—C1	119.49 (16)	C14—C9—C8	121.50 (17)
C7—C2—C1	125.73 (16)	C10—C9—C8	120.17 (17)
C13—C14—C9	121.15 (18)	C15—N1—C16	110.90 (16)
C13—C14—H14	119.4	C15—N1—C1	111.34 (14)
C9—C14—H14	119.4	C16—N1—C1	115.81 (15)
C7—C6—C5	118.07 (19)	C15—N1—H1N1	108.8 (14)
C7—C6—H6	121.0	C16—N1—H1N1	104.7 (13)
C5—C6—H6	121.0	C1—N1—H1N1	104.7 (14)
C11—C10—C9	120.47 (18)	N1—C15—H15A	109.5
C11—C10—H10	119.8	N1—C15—H15B	109.5
C9—C10—H10	119.8	H15A—C15—H15B	109.5
C6—C5—C4	120.89 (18)	N1—C15—H15C	109.5
C6—C5—H5	119.6	H15A—C15—H15C	109.5
C4—C5—H5	119.6	H15B—C15—H15C	109.5
C13—C12—C11	119.35 (19)	N1—C16—H16A	109.5
C13—C12—H12	120.3	N1—C16—H16B	109.5
C11—C12—H12	120.3	H16A—C16—H16B	109.5
C12—C11—C10	120.54 (18)	N1—C16—H16C	109.5
C12—C11—H11	119.7	H16A—C16—H16C	109.5
C10—C11—H11	119.7	H16B—C16—H16C	109.5

### [1-(2,6-Difluorophenyl)-2-phenylethyl]dimethylazanium chloride (I) Hydrogen-bond geometry (Å, º)

**Table:**

*D*—H···*A*	*D*—H	H···*A*	*D*···*A*	*D*—H···*A*
N1—H1*N*1···Cl1	0.93 (2)	2.08 (2)	3.0006 (17)	167.3 (19)

### 1-[1-(2,6-Difluorophenyl)-2-phenylethyl]pyrrolidin-1-ium chloride (IV) Crystal data

**Table:**

C_18_H_20_F_2_N^+^·Cl^−^	*Z* = 4
*M**_r_* = 323.80	*F*(000) = 680
Triclinic, *P*1	*D*_x_ = 1.308 Mg m^−^^3^
*a* = 8.1365 (4) Å	Mo *K*α radiation, λ = 0.71073 Å
*b* = 12.7421 (10) Å	Cell parameters from 9872 reflections
*c* = 16.0451 (8) Å	θ = 3.0–25.4°
α = 88.059 (5)°	µ = 0.25 mm^−^^1^
β = 82.349 (4)°	*T* = 123 K
γ = 86.140 (5)°	Block, colourless
*V* = 1644.42 (17) Å^3^	0.5 × 0.4 × 0.3 mm

### 1-[1-(2,6-Difluorophenyl)-2-phenylethyl]pyrrolidin-1-ium chloride (IV) Data collection

**Table:**

Oxford Diffraction Xcalibur diffractometer	4892 reflections with *I* > 2σ(*I*)
Radiation source: fine-focus sealed tube	*R*_int_ = 0.032
scans in phi and ω	θ_max_ = 25.0°, θ_min_ = 3.0°
Absorption correction: analytical (SADABS; Krause *et al.*, 2015)	*h* = −9→8
*T*_min_ = 0.888, *T*_max_ = 0.928	*k* = −15→15
13277 measured reflections	*l* = −19→19
5775 independent reflections	

### 1-[1-(2,6-Difluorophenyl)-2-phenylethyl]pyrrolidin-1-ium chloride (IV) Refinement

**Table:**

Refinement on *F*^2^	2 restraints
Least-squares matrix: full	Hydrogen site location: mixed
*R*[*F*^2^ > 2σ(*F*^2^)] = 0.090	H atoms treated by a mixture of independent and constrained refinement
*wR*(*F*^2^) = 0.206	*w* = 1/[σ^2^(*F*_o_^2^) + (0.0143*P*)^2^ + 10.4102*P*] where *P* = (*F*_o_^2^ + 2*F*_c_^2^)/3
*S* = 1.19	(Δ/σ)_max_ < 0.001
5775 reflections	Δρ_max_ = 0.59 e Å^−^^3^
403 parameters	Δρ_min_ = −0.34 e Å^−^^3^

### 1-[1-(2,6-Difluorophenyl)-2-phenylethyl]pyrrolidin-1-ium chloride (IV) Special details

**Table:**

Geometry. All esds (except the esd in the dihedral angle between two l.s. planes) are estimated using the full covariance matrix. The cell esds are taken into account individually in the estimation of esds in distances, angles and torsion angles; correlations between esds in cell parameters are only used when they are defined by crystal symmetry. An approximate (isotropic) treatment of cell esds is used for estimating esds involving l.s. planes.

### 1-[1-(2,6-Difluorophenyl)-2-phenylethyl]pyrrolidin-1-ium chloride (IV) Fractional atomic coordinates and isotropic or equivalent isotropic displacement parameters (Å^2^)

**Table:**

	*x*	*y*	*z*	*U*_iso_*/*U*_eq_	
Cl2	1.11424 (18)	0.86611 (11)	0.52129 (8)	0.0292 (3)	
Cl1	0.6097 (2)	0.63420 (11)	1.00579 (9)	0.0366 (4)	
F1	0.3393 (4)	0.7014 (3)	0.6933 (2)	0.0340 (8)	
F2	0.0752 (4)	0.4329 (3)	0.8568 (2)	0.0335 (8)	
F3	0.9182 (4)	0.7720 (3)	0.2168 (2)	0.0339 (8)	
F4	0.5878 (4)	1.0740 (3)	0.3087 (2)	0.0396 (9)	
N2	0.8306 (6)	0.8731 (4)	0.4140 (3)	0.0242 (10)	
N1	0.3190 (6)	0.6384 (4)	0.9063 (3)	0.0244 (10)	
C27	0.7599 (6)	0.9234 (4)	0.2683 (3)	0.0201 (11)	
C9	0.2164 (6)	0.5657 (4)	0.7790 (3)	0.0205 (11)	
C28	0.6271 (7)	0.9934 (4)	0.2546 (3)	0.0251 (12)	
C1	0.3471 (6)	0.5569 (4)	0.8386 (3)	0.0212 (11)	
H1	0.335348	0.487106	0.868486	0.025*	
C21	1.0944 (6)	1.0169 (4)	0.2351 (3)	0.0227 (11)	
C19	0.8667 (6)	0.9430 (4)	0.3359 (3)	0.0218 (11)	
H19	0.836336	1.016817	0.354039	0.026*	
C3	0.5585 (6)	0.4696 (4)	0.7320 (3)	0.0244 (12)	
C20	1.0529 (7)	0.9374 (4)	0.3056 (3)	0.0252 (12)	
H20A	1.113949	0.951212	0.353117	0.030*	
H20B	1.088997	0.865768	0.285632	0.030*	
C14	0.0907 (7)	0.4965 (4)	0.7874 (3)	0.0252 (12)	
C32	0.7907 (7)	0.8440 (4)	0.2094 (3)	0.0258 (12)	
C11	0.1145 (8)	0.6272 (5)	0.6475 (4)	0.0325 (14)	
H11	0.122464	0.673672	0.599757	0.039*	
C22	1.1007 (7)	1.1225 (5)	0.2533 (4)	0.0309 (13)	
H22	1.075832	1.144702	0.309803	0.037*	
C2	0.5263 (7)	0.5557 (4)	0.7956 (3)	0.0242 (12)	
H2A	0.546795	0.624627	0.767039	0.029*	
H2B	0.603436	0.544026	0.838268	0.029*	
C25	1.1662 (7)	1.0602 (5)	0.0882 (4)	0.0327 (14)	
H25	1.187292	1.039035	0.031390	0.039*	
C10	0.2208 (7)	0.6305 (4)	0.7066 (3)	0.0239 (12)	
C8	0.5647 (7)	0.3644 (5)	0.7585 (4)	0.0321 (13)	
H8	0.546327	0.346785	0.816886	0.038*	
C36	0.6658 (7)	0.8977 (5)	0.4644 (4)	0.0348 (14)	
H36A	0.575698	0.900454	0.428380	0.042*	
H36B	0.663620	0.965988	0.492346	0.042*	
C15	0.1649 (8)	0.6286 (5)	0.9678 (4)	0.0351 (14)	
H15A	0.174375	0.564876	1.004224	0.042*	
H15B	0.065945	0.625638	0.938275	0.042*	
C33	0.8366 (8)	0.7563 (5)	0.4037 (4)	0.0346 (14)	
H33A	0.952397	0.726924	0.389866	0.042*	
H33B	0.771135	0.738088	0.359065	0.042*	
C26	1.1262 (7)	0.9866 (4)	0.1518 (3)	0.0256 (12)	
H26	1.120489	0.914988	0.138423	0.031*	
C31	0.6991 (8)	0.8323 (5)	0.1447 (4)	0.0346 (14)	
H31	0.722886	0.774732	0.107981	0.042*	
C13	−0.0197 (7)	0.4882 (5)	0.7300 (4)	0.0338 (14)	
H13	−0.103175	0.438879	0.738846	0.041*	
C5	0.6205 (8)	0.4144 (6)	0.5880 (4)	0.0428 (16)	
H5	0.638539	0.431937	0.529606	0.051*	
C29	0.5330 (8)	0.9887 (5)	0.1896 (4)	0.0359 (15)	
H29	0.445057	1.039810	0.182690	0.043*	
C17	0.2484 (8)	0.8097 (5)	0.9603 (4)	0.0394 (15)	
H17A	0.172625	0.871072	0.948940	0.047*	
H17B	0.342017	0.834403	0.986302	0.047*	
C30	0.5719 (8)	0.9062 (6)	0.1348 (4)	0.0392 (16)	
H30	0.509337	0.900738	0.089399	0.047*	
C7	0.5979 (7)	0.2849 (5)	0.7000 (4)	0.0380 (15)	
H7	0.599135	0.213208	0.718453	0.046*	
C24	1.1755 (8)	1.1644 (5)	0.1071 (4)	0.0386 (15)	
H24	1.204076	1.214593	0.063287	0.046*	
C23	1.1434 (8)	1.1956 (5)	0.1892 (4)	0.0361 (14)	
H23	1.150355	1.267240	0.202027	0.043*	
C18	0.3116 (8)	0.7526 (4)	0.8797 (4)	0.0329 (14)	
H18A	0.234606	0.766623	0.837207	0.040*	
H18B	0.423028	0.774539	0.855950	0.040*	
C12	−0.0056 (8)	0.5533 (5)	0.6597 (4)	0.0390 (15)	
H12	−0.078813	0.547876	0.618826	0.047*	
C4	0.5858 (7)	0.4928 (5)	0.6459 (4)	0.0312 (13)	
H4	0.580421	0.564153	0.626864	0.037*	
C6	0.6290 (8)	0.3101 (6)	0.6148 (5)	0.0487 (19)	
H6	0.656059	0.256025	0.575020	0.058*	
C35	0.6475 (9)	0.8081 (6)	0.5286 (4)	0.0473 (18)	
H35A	0.530490	0.788934	0.539572	0.057*	
H35B	0.683247	0.827842	0.582205	0.057*	
C16	0.1547 (9)	0.7276 (5)	1.0185 (4)	0.0420 (16)	
H16A	0.207772	0.713990	1.070222	0.050*	
H16B	0.037391	0.752837	1.034725	0.050*	
C34	0.7597 (9)	0.7161 (6)	0.4895 (4)	0.0449 (17)	
H34A	0.847256	0.694674	0.525112	0.054*	
H34B	0.693919	0.654893	0.483513	0.054*	
H1'	0.397 (7)	0.632 (5)	0.948 (3)	0.054*	
H2	0.911 (6)	0.875 (5)	0.454 (3)	0.054*	

### 1-[1-(2,6-Difluorophenyl)-2-phenylethyl]pyrrolidin-1-ium chloride (IV) Atomic displacement parameters (Å^2^)

**Table:**

	*U*^11^	*U*^22^	*U*^33^	*U*^12^	*U*^13^	*U*^23^
Cl2	0.0390 (8)	0.0279 (7)	0.0220 (7)	0.0035 (6)	−0.0099 (6)	−0.0062 (5)
Cl1	0.0553 (10)	0.0307 (8)	0.0290 (8)	−0.0163 (7)	−0.0203 (7)	0.0075 (6)
F1	0.041 (2)	0.0338 (19)	0.0285 (18)	−0.0134 (15)	−0.0076 (15)	0.0094 (14)
F2	0.0333 (19)	0.0309 (18)	0.0360 (19)	−0.0100 (14)	−0.0017 (15)	0.0077 (15)
F3	0.038 (2)	0.0304 (18)	0.0321 (19)	0.0034 (15)	−0.0013 (15)	−0.0111 (14)
F4	0.034 (2)	0.0281 (18)	0.057 (2)	0.0064 (15)	−0.0083 (17)	−0.0129 (16)
N2	0.028 (3)	0.027 (2)	0.016 (2)	−0.003 (2)	−0.0015 (19)	0.0012 (19)
N1	0.031 (3)	0.027 (2)	0.016 (2)	−0.003 (2)	−0.0052 (19)	−0.0038 (19)
C27	0.023 (3)	0.021 (3)	0.017 (3)	−0.004 (2)	−0.002 (2)	0.000 (2)
C9	0.022 (3)	0.021 (3)	0.018 (3)	0.005 (2)	−0.002 (2)	−0.005 (2)
C28	0.023 (3)	0.025 (3)	0.027 (3)	−0.006 (2)	−0.002 (2)	−0.001 (2)
C1	0.026 (3)	0.020 (3)	0.018 (3)	−0.001 (2)	−0.006 (2)	−0.002 (2)
C21	0.015 (3)	0.029 (3)	0.024 (3)	−0.001 (2)	−0.001 (2)	−0.001 (2)
C19	0.026 (3)	0.021 (3)	0.018 (3)	−0.005 (2)	−0.003 (2)	0.002 (2)
C3	0.020 (3)	0.031 (3)	0.023 (3)	−0.003 (2)	−0.001 (2)	−0.005 (2)
C20	0.026 (3)	0.029 (3)	0.022 (3)	−0.004 (2)	−0.006 (2)	−0.001 (2)
C14	0.023 (3)	0.023 (3)	0.030 (3)	0.000 (2)	−0.003 (2)	−0.002 (2)
C32	0.027 (3)	0.027 (3)	0.022 (3)	−0.006 (2)	0.002 (2)	−0.001 (2)
C11	0.043 (4)	0.033 (3)	0.024 (3)	0.005 (3)	−0.015 (3)	0.001 (2)
C22	0.031 (3)	0.034 (3)	0.028 (3)	−0.006 (3)	−0.002 (3)	−0.006 (3)
C2	0.023 (3)	0.028 (3)	0.022 (3)	−0.006 (2)	−0.005 (2)	−0.003 (2)
C25	0.032 (3)	0.044 (4)	0.021 (3)	−0.005 (3)	0.002 (2)	0.001 (3)
C10	0.024 (3)	0.023 (3)	0.025 (3)	−0.004 (2)	−0.002 (2)	−0.001 (2)
C8	0.020 (3)	0.039 (3)	0.037 (3)	0.002 (2)	−0.003 (2)	−0.002 (3)
C36	0.027 (3)	0.053 (4)	0.024 (3)	−0.008 (3)	0.004 (2)	−0.004 (3)
C15	0.038 (4)	0.035 (3)	0.029 (3)	0.001 (3)	0.008 (3)	−0.004 (3)
C33	0.045 (4)	0.032 (3)	0.028 (3)	−0.007 (3)	−0.007 (3)	0.005 (3)
C26	0.023 (3)	0.031 (3)	0.024 (3)	−0.005 (2)	−0.003 (2)	−0.001 (2)
C31	0.036 (3)	0.044 (4)	0.025 (3)	−0.020 (3)	0.003 (3)	−0.011 (3)
C13	0.025 (3)	0.029 (3)	0.049 (4)	−0.007 (2)	−0.010 (3)	0.000 (3)
C5	0.038 (4)	0.063 (5)	0.026 (3)	0.002 (3)	0.004 (3)	−0.015 (3)
C29	0.030 (3)	0.038 (4)	0.042 (4)	−0.008 (3)	−0.016 (3)	0.011 (3)
C17	0.044 (4)	0.032 (3)	0.043 (4)	0.005 (3)	−0.007 (3)	−0.016 (3)
C30	0.035 (4)	0.062 (4)	0.026 (3)	−0.022 (3)	−0.013 (3)	0.003 (3)
C7	0.027 (3)	0.035 (3)	0.050 (4)	0.000 (3)	0.002 (3)	−0.011 (3)
C24	0.037 (4)	0.047 (4)	0.031 (3)	−0.007 (3)	−0.002 (3)	0.015 (3)
C23	0.034 (3)	0.026 (3)	0.048 (4)	−0.003 (3)	−0.002 (3)	−0.001 (3)
C18	0.046 (4)	0.022 (3)	0.031 (3)	−0.001 (3)	−0.005 (3)	−0.005 (2)
C12	0.033 (3)	0.041 (4)	0.049 (4)	0.000 (3)	−0.024 (3)	−0.005 (3)
C4	0.029 (3)	0.038 (3)	0.026 (3)	−0.004 (3)	0.000 (2)	−0.003 (3)
C6	0.031 (4)	0.057 (5)	0.056 (5)	0.000 (3)	0.008 (3)	−0.034 (4)
C35	0.045 (4)	0.075 (5)	0.023 (3)	−0.020 (4)	0.001 (3)	0.003 (3)
C16	0.046 (4)	0.047 (4)	0.031 (3)	0.004 (3)	0.001 (3)	−0.012 (3)
C34	0.047 (4)	0.056 (4)	0.034 (4)	−0.015 (3)	−0.009 (3)	0.018 (3)

### 1-[1-(2,6-Difluorophenyl)-2-phenylethyl]pyrrolidin-1-ium chloride (IV) Geometric parameters (Å, º)

**Table:**

F1—C10	1.357 (6)	C3—C4	1.393 (8)
F2—C14	1.352 (6)	C3—C2	1.510 (7)
F3—C32	1.353 (6)	C14—C13	1.381 (8)
F4—C28	1.361 (6)	C32—C31	1.373 (8)
N2—C36	1.491 (7)	C11—C10	1.369 (8)
N2—C33	1.500 (7)	C11—C12	1.393 (9)
N2—C19	1.520 (6)	C22—C23	1.389 (8)
N1—C15	1.498 (7)	C25—C24	1.381 (9)
N1—C18	1.502 (7)	C25—C26	1.383 (8)
N1—C1	1.513 (6)	C8—C7	1.394 (8)
C27—C28	1.390 (7)	C36—C35	1.513 (9)
C27—C32	1.396 (7)	C15—C16	1.515 (8)
C27—C19	1.513 (7)	C33—C34	1.521 (8)
C9—C14	1.385 (7)	C31—C30	1.373 (9)
C9—C10	1.400 (7)	C13—C12	1.374 (9)
C9—C1	1.518 (7)	C5—C4	1.378 (9)
C28—C29	1.378 (8)	C5—C6	1.382 (10)
C1—C2	1.528 (7)	C29—C30	1.386 (9)
C21—C26	1.391 (7)	C17—C18	1.516 (8)
C21—C22	1.392 (8)	C17—C16	1.550 (9)
C21—C20	1.513 (7)	C7—C6	1.388 (10)
C19—C20	1.526 (7)	C24—C23	1.375 (9)
C3—C8	1.392 (8)	C35—C34	1.534 (10)
			
C36—N2—C33	103.9 (4)	F3—C32—C31	117.2 (5)
C36—N2—C19	114.4 (4)	F3—C32—C27	118.1 (5)
C33—N2—C19	118.5 (4)	C31—C32—C27	124.8 (5)
C15—N1—C18	103.7 (4)	C10—C11—C12	117.7 (5)
C15—N1—C1	115.1 (4)	C23—C22—C21	120.2 (5)
C18—N1—C1	118.3 (4)	C3—C2—C1	110.2 (4)
C28—C27—C32	113.3 (5)	C24—C25—C26	120.2 (6)
C28—C27—C19	120.6 (5)	F1—C10—C11	117.3 (5)
C32—C27—C19	125.7 (5)	F1—C10—C9	118.0 (5)
C14—C9—C10	113.8 (5)	C11—C10—C9	124.7 (5)
C14—C9—C1	120.1 (5)	C3—C8—C7	120.4 (6)
C10—C9—C1	125.5 (5)	N2—C36—C35	104.2 (5)
F4—C28—C29	117.1 (5)	N1—C15—C16	103.7 (5)
F4—C28—C27	117.7 (5)	N2—C33—C34	103.2 (5)
C29—C28—C27	125.2 (5)	C25—C26—C21	120.3 (5)
N1—C1—C9	113.3 (4)	C30—C31—C32	118.0 (6)
N1—C1—C2	110.0 (4)	C12—C13—C14	118.2 (5)
C9—C1—C2	114.7 (4)	C4—C5—C6	120.0 (6)
C26—C21—C22	119.0 (5)	C28—C29—C30	117.2 (6)
C26—C21—C20	121.1 (5)	C18—C17—C16	105.3 (5)
C22—C21—C20	119.9 (5)	C31—C30—C29	121.4 (5)
C27—C19—N2	113.2 (4)	C6—C7—C8	120.1 (6)
C27—C19—C20	114.3 (4)	C23—C24—C25	120.0 (6)
N2—C19—C20	110.0 (4)	C24—C23—C22	120.2 (6)
C8—C3—C4	118.4 (5)	N1—C18—C17	104.2 (5)
C8—C3—C2	120.3 (5)	C13—C12—C11	121.0 (5)
C4—C3—C2	121.3 (5)	C5—C4—C3	121.4 (6)
C21—C20—C19	111.0 (4)	C5—C6—C7	119.7 (6)
F2—C14—C13	117.9 (5)	C36—C35—C34	105.5 (5)
F2—C14—C9	117.6 (5)	C15—C16—C17	105.7 (5)
C13—C14—C9	124.6 (5)	C33—C34—C35	105.9 (5)

### 1-[1-(2,6-Difluorophenyl)-2-phenylethyl]pyrrolidin-1-ium chloride (IV) Hydrogen-bond geometry (Å, º)

**Table:**

*D*—H···*A*	*D*—H	H···*A*	*D*···*A*	*D*—H···*A*
N1—H1′···Cl1	0.99 (1)	2.07 (2)	3.021 (5)	163 (6)
N2—H2···Cl2	0.98 (1)	2.08 (2)	3.052 (5)	169 (6)

### [1-(2,6-Difluorophenyl)-2-phenylethyl](ethyl)azanium chloride dichloromethane hemisolvate (II) Crystal data

**Table:**

2C_16_H_18_F_2_N^+^·2Cl^−^·CH_2_Cl_2_	*F*(000) = 1416
*M**_r_* = 680.45	*D*_x_ = 1.313 Mg m^−^^3^
Monoclinic, *I*2/*a*	Mo *K*α radiation, λ = 0.71073 Å
*a* = 22.9963 (14) Å	Cell parameters from 8967 reflections
*b* = 7.8729 (5) Å	θ = 3.0–26.4°
*c* = 19.033 (1) Å	µ = 0.39 mm^−^^1^
β = 92.130 (5)°	*T* = 123 K
*V* = 3443.5 (4) Å^3^	Block, colourless
*Z* = 4	0.3 × 0.2 × 0.1 mm

### [1-(2,6-Difluorophenyl)-2-phenylethyl](ethyl)azanium chloride dichloromethane hemisolvate (II) Data collection

**Table:**

Oxford Diffraction Xcalibur diffractometer	2813 reflections with *I* > 2σ(*I*)
Radiation source: fine-focus sealed tube	*R*_int_ = 0.021
scans in phi and ω	θ_max_ = 25.0°, θ_min_ = 3.3°
Absorption correction: analytical (SADABS; Krause *et al.*, 2015)	*h* = −27→26
*T*_min_ = 0.911, *T*_max_ = 0.962	*k* = −9→9
12738 measured reflections	*l* = −21→22
3034 independent reflections	

### [1-(2,6-Difluorophenyl)-2-phenylethyl](ethyl)azanium chloride dichloromethane hemisolvate (II) Refinement

**Table:**

Refinement on *F*^2^	0 restraints
Least-squares matrix: full	Hydrogen site location: mixed
*R*[*F*^2^ > 2σ(*F*^2^)] = 0.031	H atoms treated by a mixture of independent and constrained refinement
*wR*(*F*^2^) = 0.077	*w* = 1/[σ^2^(*F*_o_^2^) + (0.0343*P*)^2^ + 2.8401*P*] where *P* = (*F*_o_^2^ + 2*F*_c_^2^)/3
*S* = 1.07	(Δ/σ)_max_ = 0.001
3034 reflections	Δρ_max_ = 0.25 e Å^−^^3^
224 parameters	Δρ_min_ = −0.22 e Å^−^^3^

### [1-(2,6-Difluorophenyl)-2-phenylethyl](ethyl)azanium chloride dichloromethane hemisolvate (II) Special details

**Table:**

Geometry. All esds (except the esd in the dihedral angle between two l.s. planes) are estimated using the full covariance matrix. The cell esds are taken into account individually in the estimation of esds in distances, angles and torsion angles; correlations between esds in cell parameters are only used when they are defined by crystal symmetry. An approximate (isotropic) treatment of cell esds is used for estimating esds involving l.s. planes.

### [1-(2,6-Difluorophenyl)-2-phenylethyl](ethyl)azanium chloride dichloromethane hemisolvate (II) Fractional atomic coordinates and isotropic or equivalent isotropic displacement parameters (Å^2^)

**Table:**

	*x*	*y*	*z*	*U*_iso_*/*U*_eq_	Occ. (<1)
Cl1	0.28641 (2)	0.60990 (5)	0.16223 (2)	0.02972 (12)	
Cl1S	0.31326 (2)	0.28821 (7)	0.00859 (3)	0.05486 (16)	
F1	0.05520 (5)	1.14129 (13)	0.11535 (6)	0.0466 (3)	
F2	0.17656 (5)	0.69870 (15)	0.04908 (5)	0.0570 (3)	
N1	0.19574 (5)	0.90437 (17)	0.17878 (7)	0.0248 (3)	
C1	0.13169 (6)	0.88167 (19)	0.16264 (7)	0.0251 (3)	
H1	0.110554	0.967062	0.191048	0.030*	
C9	0.11629 (6)	0.91562 (18)	0.08602 (7)	0.0242 (3)	
C3	0.05036 (7)	0.6689 (2)	0.17558 (8)	0.0308 (3)	
C13	0.12414 (8)	0.8489 (2)	−0.03848 (9)	0.0391 (4)	
H13	0.140233	0.779839	−0.073793	0.047*	
C12	0.08611 (7)	0.9784 (2)	−0.05562 (9)	0.0392 (4)	
H12	0.075593	0.999429	−0.103567	0.047*	
C11	0.06315 (7)	1.0777 (2)	−0.00420 (9)	0.0369 (4)	
H11	0.037235	1.168104	−0.016069	0.044*	
C14	0.13806 (7)	0.8227 (2)	0.03156 (8)	0.0326 (4)	
C10	0.07854 (6)	1.04341 (19)	0.06485 (8)	0.0288 (3)	
C4	0.00903 (8)	0.7455 (2)	0.21652 (9)	0.0399 (4)	
H4	0.021169	0.820557	0.253303	0.048*	
C15	0.2196 (4)	1.0751 (12)	0.1558 (4)	0.0316 (19)	0.707 (5)
H15A	0.195508	1.167814	0.174557	0.038*	0.707 (5)
H15B	0.217609	1.082569	0.103855	0.038*	0.707 (5)
C8	0.03174 (8)	0.5597 (2)	0.12252 (9)	0.0388 (4)	
H8	0.059436	0.506416	0.093985	0.047*	
C2	0.11439 (7)	0.7045 (2)	0.18834 (9)	0.0326 (4)	
H2A	0.137251	0.617767	0.163763	0.039*	
H2B	0.124134	0.695173	0.239296	0.039*	
C7	−0.02704 (9)	0.5276 (3)	0.11066 (10)	0.0501 (5)	
H7	−0.039328	0.451887	0.074176	0.060*	
C5	−0.04964 (8)	0.7136 (3)	0.20423 (11)	0.0490 (5)	
H5	−0.077544	0.767277	0.232379	0.059*	
C6	−0.06771 (8)	0.6042 (3)	0.15123 (11)	0.0524 (5)	
H6	−0.107970	0.581969	0.142816	0.063*	
C16	0.28347 (10)	1.0963 (3)	0.18310 (13)	0.0360 (7)	0.707 (5)
H16A	0.285642	1.081935	0.234280	0.054*	0.707 (5)
H16B	0.297386	1.209892	0.171032	0.054*	0.707 (5)
H16C	0.307818	1.010445	0.161259	0.054*	0.707 (5)
C1S	0.250000	0.4136 (3)	0.000000	0.0302 (5)	
H1S1	0.252617	0.487565	−0.041826	0.036*	0.5
H1S2	0.247383	0.487565	0.041827	0.036*	0.5
C16A	0.1932 (3)	1.2115 (7)	0.1843 (3)	0.0383 (18)	0.293 (5)
H16D	0.155523	1.220984	0.158750	0.057*	0.293 (5)
H16E	0.216492	1.313058	0.175641	0.057*	0.293 (5)
H16F	0.186879	1.201409	0.234797	0.057*	0.293 (5)
H1B	0.2162 (8)	0.819 (2)	0.1612 (9)	0.036 (5)*	
H1A	0.2010 (7)	0.893 (2)	0.2273 (10)	0.034 (5)*	
C15A	0.2248 (10)	1.057 (3)	0.1594 (10)	0.037 (6)	0.293 (5)
H15C	0.227354	1.060956	0.107617	0.044*	0.293 (5)
H15D	0.264950	1.056010	0.180153	0.044*	0.293 (5)

### [1-(2,6-Difluorophenyl)-2-phenylethyl](ethyl)azanium chloride dichloromethane hemisolvate (II) Atomic displacement parameters (Å^2^)

**Table:**

	*U*^11^	*U*^22^	*U*^33^	*U*^12^	*U*^13^	*U*^23^
Cl1	0.0298 (2)	0.0380 (2)	0.02123 (18)	0.00784 (16)	0.00001 (14)	−0.00054 (15)
Cl1S	0.0491 (3)	0.0558 (3)	0.0581 (3)	0.0262 (2)	−0.0191 (2)	−0.0251 (2)
F1	0.0486 (6)	0.0405 (6)	0.0507 (6)	0.0214 (5)	0.0023 (5)	−0.0013 (5)
F2	0.0669 (7)	0.0629 (7)	0.0402 (6)	0.0416 (6)	−0.0113 (5)	−0.0158 (5)
N1	0.0261 (7)	0.0268 (7)	0.0211 (7)	0.0025 (6)	−0.0028 (5)	0.0011 (6)
C1	0.0246 (7)	0.0264 (8)	0.0239 (7)	0.0019 (6)	−0.0022 (6)	0.0004 (6)
C9	0.0217 (7)	0.0253 (8)	0.0254 (7)	−0.0011 (6)	−0.0027 (6)	0.0023 (6)
C3	0.0340 (8)	0.0268 (8)	0.0313 (8)	−0.0038 (7)	−0.0044 (7)	0.0103 (7)
C13	0.0366 (9)	0.0524 (11)	0.0280 (8)	−0.0036 (8)	−0.0014 (7)	−0.0061 (8)
C12	0.0325 (9)	0.0556 (11)	0.0287 (8)	−0.0121 (8)	−0.0080 (7)	0.0119 (8)
C11	0.0271 (8)	0.0381 (9)	0.0448 (10)	−0.0028 (7)	−0.0082 (7)	0.0179 (8)
C14	0.0301 (8)	0.0358 (9)	0.0314 (8)	0.0074 (7)	−0.0057 (6)	−0.0028 (7)
C10	0.0242 (7)	0.0266 (8)	0.0356 (8)	0.0002 (6)	0.0009 (6)	0.0031 (7)
C4	0.0433 (10)	0.0393 (10)	0.0372 (9)	−0.0060 (8)	0.0029 (8)	0.0050 (8)
C15	0.023 (2)	0.030 (3)	0.041 (3)	−0.011 (3)	−0.0100 (19)	0.014 (2)
C8	0.0451 (10)	0.0303 (9)	0.0405 (10)	−0.0053 (8)	−0.0048 (8)	0.0039 (7)
C2	0.0331 (9)	0.0294 (8)	0.0349 (9)	−0.0014 (7)	−0.0056 (7)	0.0078 (7)
C7	0.0563 (12)	0.0426 (11)	0.0499 (11)	−0.0201 (9)	−0.0178 (9)	0.0104 (9)
C5	0.0373 (10)	0.0504 (11)	0.0599 (12)	0.0000 (9)	0.0101 (9)	0.0196 (10)
C6	0.0349 (10)	0.0546 (12)	0.0665 (13)	−0.0143 (9)	−0.0130 (9)	0.0315 (11)
C16	0.0326 (14)	0.0355 (14)	0.0397 (14)	−0.0085 (10)	−0.0007 (10)	0.0018 (10)
C1S	0.0286 (11)	0.0273 (11)	0.0340 (12)	0.000	−0.0070 (9)	0.000
C16A	0.040 (3)	0.031 (3)	0.043 (3)	−0.010 (3)	−0.010 (3)	−0.002 (3)
C15A	0.053 (9)	0.042 (8)	0.015 (5)	0.024 (5)	−0.009 (4)	0.006 (5)

### [1-(2,6-Difluorophenyl)-2-phenylethyl](ethyl)azanium chloride dichloromethane hemisolvate (II) Geometric parameters (Å, º)

**Table:**

Cl1S—C1S	1.7606 (13)	C3—C2	1.510 (2)
F1—C10	1.3580 (18)	C13—C12	1.375 (3)
F2—C14	1.3516 (18)	C13—C14	1.375 (2)
N1—C15A	1.43 (2)	C12—C11	1.374 (3)
N1—C1	1.5040 (19)	C11—C10	1.375 (2)
N1—C15	1.522 (8)	C4—C5	1.384 (3)
C1—C9	1.512 (2)	C15—C16	1.550 (8)
C1—C2	1.535 (2)	C8—C7	1.385 (3)
C9—C14	1.378 (2)	C7—C6	1.374 (3)
C9—C10	1.379 (2)	C5—C6	1.379 (3)
C3—C8	1.382 (2)	C16A—C15A	1.51 (2)
C3—C4	1.389 (2)		
			
C15A—N1—C1	120.8 (11)	F2—C14—C9	116.80 (14)
C1—N1—C15	114.0 (4)	C13—C14—C9	124.89 (15)
N1—C1—C9	111.62 (12)	F1—C10—C11	118.07 (14)
N1—C1—C2	107.84 (12)	F1—C10—C9	117.92 (14)
C9—C1—C2	114.45 (13)	C11—C10—C9	124.01 (15)
C14—C9—C10	114.19 (14)	C5—C4—C3	120.64 (17)
C14—C9—C1	123.60 (13)	N1—C15—C16	110.2 (7)
C10—C9—C1	122.21 (13)	C3—C8—C7	120.44 (18)
C8—C3—C4	118.70 (16)	C3—C2—C1	112.35 (13)
C8—C3—C2	120.46 (15)	C6—C7—C8	120.56 (18)
C4—C3—C2	120.84 (15)	C6—C5—C4	120.15 (19)
C12—C13—C14	117.66 (16)	C7—C6—C5	119.51 (18)
C11—C12—C13	120.75 (15)	Cl1S—C1S—Cl1S^i^	111.79 (12)
C12—C11—C10	118.49 (15)	N1—C15A—C16A	111.2 (13)
F2—C14—C13	118.30 (14)		

Symmetry code: (i) −*x*+1/2, *y*, −*z*.

### [1-(2,6-Difluorophenyl)-2-phenylethyl](ethyl)azanium chloride dichloromethane hemisolvate (II) Hydrogen-bond geometry (Å, º)

**Table:**

*D*—H···*A*	*D*—H	H···*A*	*D*···*A*	*D*—H···*A*
N1—H1*B*···Cl1	0.89 (2)	2.30 (2)	3.1417 (14)	156.0 (16)

### tert-Butyl[1-(2,6-difluorophenyl)-2-phenylethyl]azanium chloride (III) Crystal data

**Table:**

C_18_H_22_F_2_N^+^·Cl^−^	*F*(000) = 688
*M**_r_* = 325.81	*D*_x_ = 1.270 Mg m^−^^3^
Monoclinic, *P*2_1_/*c*	Mo *K*α radiation, λ = 0.71073 Å
*a* = 11.3115 (6) Å	Cell parameters from 8922 reflections
*b* = 10.5400 (5) Å	θ = 3.0–26.2°
*c* = 14.8039 (7) Å	µ = 0.24 mm^−^^1^
β = 105.044 (5)°	*T* = 123 K
*V* = 1704.48 (15) Å^3^	Block, colourless
*Z* = 4	0.4 × 0.2 × 0.1 mm

### tert-Butyl[1-(2,6-difluorophenyl)-2-phenylethyl]azanium chloride (III) Data collection

**Table:**

Oxford Diffraction Xcalibur diffractometer	2343 reflections with *I* > 2σ(*I*)
Radiation source: fine-focus sealed tube	*R*_int_ = 0.036
scans in phi and ω	θ_max_ = 25.0°, θ_min_ = 3.3°
Absorption correction: analytical (SADABS; Krause *et al.*, 2015)	*h* = −13→13
*T*_min_ = 0.944, *T*_max_ = 0.976	*k* = −8→12
7223 measured reflections	*l* = −17→12
3008 independent reflections	

### tert-Butyl[1-(2,6-difluorophenyl)-2-phenylethyl]azanium chloride (III) Refinement

**Table:**

Refinement on *F*^2^	0 restraints
Least-squares matrix: full	Hydrogen site location: mixed
*R*[*F*^2^ > 2σ(*F*^2^)] = 0.049	H atoms treated by a mixture of independent and constrained refinement
*wR*(*F*^2^) = 0.095	*w* = 1/[σ^2^(*F*_o_^2^) + (0.0303*P*)^2^ + 0.5824*P*] where *P* = (*F*_o_^2^ + 2*F*_c_^2^)/3
*S* = 1.06	(Δ/σ)_max_ < 0.001
3008 reflections	Δρ_max_ = 0.22 e Å^−^^3^
210 parameters	Δρ_min_ = −0.25 e Å^−^^3^

### tert-Butyl[1-(2,6-difluorophenyl)-2-phenylethyl]azanium chloride (III) Special details

**Table:**

Geometry. All esds (except the esd in the dihedral angle between two l.s. planes) are estimated using the full covariance matrix. The cell esds are taken into account individually in the estimation of esds in distances, angles and torsion angles; correlations between esds in cell parameters are only used when they are defined by crystal symmetry. An approximate (isotropic) treatment of cell esds is used for estimating esds involving l.s. planes.

### tert-Butyl[1-(2,6-difluorophenyl)-2-phenylethyl]azanium chloride (III) Fractional atomic coordinates and isotropic or equivalent isotropic displacement parameters (Å^2^)

**Table:**

	*x*	*y*	*z*	*U*_iso_*/*U*_eq_	
Cl1	0.39037 (5)	1.13981 (5)	0.05585 (4)	0.02430 (17)	
F1	0.09857 (12)	0.74034 (14)	0.10106 (9)	0.0339 (4)	
F2	0.39994 (13)	0.60187 (13)	−0.04167 (9)	0.0323 (4)	
N1	0.41820 (17)	0.84575 (19)	0.06664 (12)	0.0173 (4)	
C1	0.5951 (2)	0.8889 (2)	0.19595 (15)	0.0253 (6)	
H1C	0.648652	0.862937	0.156776	0.038*	
H1D	0.636607	0.873592	0.261856	0.038*	
H1E	0.576024	0.979356	0.186458	0.038*	
C2	0.3904 (2)	0.8518 (2)	0.22761 (15)	0.0266 (6)	
H2A	0.367671	0.941068	0.215264	0.040*	
H2B	0.431267	0.840815	0.294127	0.040*	
H2C	0.316581	0.799009	0.211062	0.040*	
C3	0.5069 (2)	0.6716 (2)	0.18050 (15)	0.0264 (6)	
H3A	0.430789	0.622529	0.168216	0.040*	
H3B	0.555718	0.655092	0.244475	0.040*	
H3C	0.553596	0.646429	0.136139	0.040*	
C4	0.4769 (2)	0.8122 (2)	0.16915 (14)	0.0193 (5)	
C5	0.2864 (2)	0.8140 (2)	0.02029 (14)	0.0186 (5)	
H5	0.234618	0.867019	0.050999	0.022*	
C6	0.2527 (2)	0.6777 (2)	0.03035 (14)	0.0185 (5)	
C7	0.1596 (2)	0.6448 (2)	0.07147 (15)	0.0233 (5)	
C8	0.1273 (2)	0.5219 (3)	0.08481 (17)	0.0325 (6)	
H8	0.064723	0.504267	0.115178	0.039*	
C9	0.1874 (2)	0.4250 (3)	0.05328 (17)	0.0352 (7)	
H9	0.166274	0.339447	0.061977	0.042*	
C10	0.2784 (2)	0.4506 (2)	0.00904 (17)	0.0313 (6)	
H10	0.319903	0.384076	−0.013306	0.038*	
C11	0.3068 (2)	0.5751 (2)	−0.00151 (15)	0.0234 (6)	
C12	0.2600 (2)	0.8573 (2)	−0.08193 (14)	0.0213 (5)	
H12A	0.290438	0.944996	−0.084222	0.026*	
H12B	0.304422	0.801691	−0.115937	0.026*	
C13	0.1247 (2)	0.8529 (2)	−0.12948 (14)	0.0207 (5)	
C14	0.0489 (2)	0.9493 (3)	−0.11500 (17)	0.0332 (6)	
H14	0.082380	1.018435	−0.075390	0.040*	
C15	−0.0752 (3)	0.9463 (3)	−0.15751 (19)	0.0444 (8)	
H15	−0.126360	1.013158	−0.147073	0.053*	
C16	−0.1250 (2)	0.8465 (3)	−0.21498 (19)	0.0415 (7)	
H16	−0.210343	0.844521	−0.244199	0.050*	
C17	−0.0509 (2)	0.7503 (3)	−0.22978 (17)	0.0368 (7)	
H17	−0.084782	0.681460	−0.269479	0.044*	
C18	0.0738 (2)	0.7533 (2)	−0.18684 (15)	0.0274 (6)	
H18	0.124631	0.685936	−0.197099	0.033*	
H1A	0.423 (2)	0.933 (2)	0.0648 (15)	0.020 (6)*	
H1B	0.471 (2)	0.818 (2)	0.0302 (16)	0.033 (7)*	

### tert-Butyl[1-(2,6-difluorophenyl)-2-phenylethyl]azanium chloride (III) Atomic displacement parameters (Å^2^)

**Table:**

	*U*^11^	*U*^22^	*U*^33^	*U*^12^	*U*^13^	*U*^23^
Cl1	0.0305 (3)	0.0186 (3)	0.0261 (3)	0.0042 (3)	0.0115 (2)	0.0024 (3)
F1	0.0296 (8)	0.0364 (9)	0.0419 (8)	0.0001 (7)	0.0202 (7)	0.0009 (7)
F2	0.0383 (9)	0.0276 (8)	0.0363 (8)	0.0065 (7)	0.0190 (7)	0.0004 (7)
N1	0.0222 (11)	0.0132 (11)	0.0172 (10)	0.0009 (10)	0.0063 (8)	0.0011 (9)
C1	0.0248 (14)	0.0286 (15)	0.0205 (12)	−0.0010 (12)	0.0021 (10)	0.0037 (11)
C2	0.0302 (14)	0.0320 (15)	0.0186 (12)	−0.0025 (13)	0.0081 (10)	−0.0008 (11)
C3	0.0329 (15)	0.0232 (14)	0.0205 (12)	0.0024 (12)	0.0021 (10)	0.0061 (11)
C4	0.0213 (13)	0.0210 (12)	0.0142 (11)	0.0003 (11)	0.0021 (9)	0.0035 (10)
C5	0.0198 (13)	0.0168 (12)	0.0196 (12)	0.0025 (11)	0.0056 (9)	0.0006 (10)
C6	0.0197 (13)	0.0191 (12)	0.0151 (11)	0.0001 (11)	0.0019 (9)	0.0020 (10)
C7	0.0207 (13)	0.0252 (14)	0.0225 (12)	0.0007 (12)	0.0029 (10)	0.0010 (11)
C8	0.0302 (15)	0.0340 (16)	0.0317 (14)	−0.0100 (13)	0.0051 (11)	0.0075 (13)
C9	0.0429 (17)	0.0215 (15)	0.0338 (15)	−0.0113 (14)	−0.0031 (12)	0.0069 (12)
C10	0.0389 (16)	0.0182 (13)	0.0315 (14)	0.0024 (13)	−0.0003 (12)	−0.0005 (12)
C11	0.0264 (14)	0.0242 (14)	0.0198 (12)	0.0009 (12)	0.0061 (10)	0.0019 (11)
C12	0.0228 (13)	0.0209 (13)	0.0197 (11)	0.0004 (11)	0.0047 (9)	0.0041 (11)
C13	0.0241 (13)	0.0213 (13)	0.0171 (11)	−0.0003 (12)	0.0059 (9)	0.0054 (11)
C14	0.0326 (16)	0.0272 (15)	0.0347 (14)	0.0043 (13)	−0.0005 (12)	−0.0044 (12)
C15	0.0326 (17)	0.0459 (19)	0.0513 (18)	0.0166 (15)	0.0047 (13)	−0.0013 (16)
C16	0.0226 (15)	0.052 (2)	0.0457 (16)	0.0001 (15)	0.0015 (12)	0.0001 (16)
C17	0.0356 (17)	0.0408 (17)	0.0305 (14)	−0.0099 (15)	0.0024 (12)	−0.0106 (13)
C18	0.0272 (15)	0.0280 (15)	0.0278 (13)	0.0015 (12)	0.0086 (11)	−0.0018 (12)

### tert-Butyl[1-(2,6-difluorophenyl)-2-phenylethyl]azanium chloride (III) Geometric parameters (Å, º)

**Table:**

F1—C7	1.356 (3)	C6—C7	1.390 (3)
F2—C11	1.367 (2)	C7—C8	1.374 (3)
N1—C5	1.508 (3)	C8—C9	1.374 (4)
N1—C4	1.532 (3)	C8—H8	0.9500
N1—H1A	0.92 (2)	C9—C10	1.382 (4)
N1—H1B	0.95 (2)	C9—H9	0.9500
C1—C4	1.524 (3)	C10—C11	1.369 (3)
C1—H1C	0.9800	C10—H10	0.9500
C1—H1D	0.9800	C12—C13	1.510 (3)
C1—H1E	0.9800	C12—H12A	0.9900
C2—C4	1.523 (3)	C12—H12B	0.9900
C2—H2A	0.9800	C13—C18	1.379 (3)
C2—H2B	0.9800	C13—C14	1.382 (3)
C2—H2C	0.9800	C14—C15	1.381 (4)
C3—C4	1.520 (3)	C14—H14	0.9500
C3—H3A	0.9800	C15—C16	1.377 (4)
C3—H3B	0.9800	C15—H15	0.9500
C3—H3C	0.9800	C16—C17	1.369 (4)
C5—C6	1.504 (3)	C16—H16	0.9500
C5—C12	1.534 (3)	C17—C18	1.389 (3)
C5—H5	1.0000	C17—H17	0.9500
C6—C11	1.384 (3)	C18—H18	0.9500
			
C5—N1—C4	121.50 (16)	F1—C7—C8	118.5 (2)
C5—N1—H1A	105.3 (14)	F1—C7—C6	117.6 (2)
C4—N1—H1A	104.3 (14)	C8—C7—C6	123.9 (2)
C5—N1—H1B	111.4 (14)	C9—C8—C7	118.6 (2)
C4—N1—H1B	108.5 (15)	C9—C8—H8	120.7
H1A—N1—H1B	104.2 (19)	C7—C8—H8	120.7
C4—C1—H1C	109.5	C8—C9—C10	120.7 (2)
C4—C1—H1D	109.5	C8—C9—H9	119.6
H1C—C1—H1D	109.5	C10—C9—H9	119.6
C4—C1—H1E	109.5	C11—C10—C9	117.8 (2)
H1C—C1—H1E	109.5	C11—C10—H10	121.1
H1D—C1—H1E	109.5	C9—C10—H10	121.1
C4—C2—H2A	109.5	F2—C11—C10	118.5 (2)
C4—C2—H2B	109.5	F2—C11—C6	116.5 (2)
H2A—C2—H2B	109.5	C10—C11—C6	124.9 (2)
C4—C2—H2C	109.5	C13—C12—C5	111.39 (17)
H2A—C2—H2C	109.5	C13—C12—H12A	109.4
H2B—C2—H2C	109.5	C5—C12—H12A	109.4
C4—C3—H3A	109.5	C13—C12—H12B	109.4
C4—C3—H3B	109.5	C5—C12—H12B	109.4
H3A—C3—H3B	109.5	H12A—C12—H12B	108.0
C4—C3—H3C	109.5	C18—C13—C14	118.6 (2)
H3A—C3—H3C	109.5	C18—C13—C12	121.4 (2)
H3B—C3—H3C	109.5	C14—C13—C12	120.0 (2)
C3—C4—C2	111.26 (19)	C15—C14—C13	120.7 (3)
C3—C4—C1	109.42 (19)	C15—C14—H14	119.7
C2—C4—C1	110.85 (19)	C13—C14—H14	119.7
C3—C4—N1	111.22 (18)	C16—C15—C14	120.2 (3)
C2—C4—N1	108.81 (18)	C16—C15—H15	119.9
C1—C4—N1	105.11 (17)	C14—C15—H15	119.9
C6—C5—N1	114.40 (18)	C17—C16—C15	119.6 (3)
C6—C5—C12	113.12 (18)	C17—C16—H16	120.2
N1—C5—C12	107.39 (16)	C15—C16—H16	120.2
C6—C5—H5	107.2	C16—C17—C18	120.1 (3)
N1—C5—H5	107.2	C16—C17—H17	119.9
C12—C5—H5	107.2	C18—C17—H17	119.9
C11—C6—C7	114.0 (2)	C13—C18—C17	120.7 (2)
C11—C6—C5	124.53 (19)	C13—C18—H18	119.6
C7—C6—C5	121.5 (2)	C17—C18—H18	119.6

### tert-Butyl[1-(2,6-difluorophenyl)-2-phenylethyl]azanium chloride (III) Hydrogen-bond geometry (Å, º)

**Table:**

*D*—H···*A*	*D*—H	H···*A*	*D*···*A*	*D*—H···*A*
N1—H1*A*···Cl1	0.92 (2)	2.21 (3)	3.115 (2)	167.7 (19)
N1—H1*B*···Cl1^i^	0.95 (2)	2.31 (2)	3.1684 (19)	151 (2)

Symmetry code: (i) −*x*+1, −*y*+2, −*z*.
